# Solving the 3D UAV Path Planning Problem Using an Improved Multi-Leader Multi-Objective Whale Optimization Algorithm

**DOI:** 10.3390/biomimetics11070459

**Published:** 2026-07-01

**Authors:** Binbin Tu, Jiawei Bao, Haoyuan Zhou, Yan Huo, Xiaowei Han, Nanmu Hui

**Affiliations:** 1 School of Intelligent Science and Information Engineering, Shenyang University, Shenyang 110044, China; bxforever@syu.edu.cn (B.T.); syu_zhy@stu.syu.edu.cn (H.Z.); huoyandlut@hotmail.com (Y.H.); huinm@syu.edu.cn (N.H.); 2 School of Mechanical Engineering, Shenyang University, Shenyang 110044, China; hanxw@syu.edu.cn

**Keywords:** UAV path planning, multi-objective whale optimization algorithm, adaptive opposition-based learning, multi-leader mechanism, grid-based external archive, swarm intelligence

## Abstract

UAV path planning in complex static 3D environments involves multiple conflicting objectives and intricate constraints. However, when applied to highly constrained path planning tasks, MOWOA often suffers from a low proportion of feasible solutions, convergence instability, single-leader search bias, and an uneven distribution of Pareto solutions. To address these issues, this study formulates the UAV path planning problem as a multi-objective optimization problem that simultaneously considers path length, threat cost, smoothness cost, and altitude cost, and proposes an improved multi-leader multi-objective whale optimization algorithm (IML-MOWOA). The proposed IML-MOWOA progressively improves three key stages of the optimization process: initial population construction, search guidance, and external archive maintenance. Specifically, an adaptive opposition-based learning initialization strategy is first introduced to improve the feasibility and spatial coverage of initial paths. Based on the resulting non-dominated solution set, a grid-based external archive update strategy is then used to regulate solution density and provide representative candidate leaders from sparse Pareto regions. Subsequently, a multi-leader dynamic weighted search mechanism with Softmax-based cosine annealing integrates these leaders into the WOA update process, thereby enhancing multi-directional path exploration and alleviating premature convergence. Comparative experiments conducted in three static 3D environments of varying complexity demonstrate that the proposed method achieves more robust convergence, better Pareto-front distribution, and more balanced task-level path quality than the benchmark algorithms. In the most challenging scenario, IML-MOWOA achieves the highest number of feasible paths, reduces the mean IGD by 25.04%, and decreases the mean path length, threat cost, smoothness cost, and altitude cost by 1.65%, 28.45%, 53.23%, and 29.88%, respectively, compared with the best-performing competing algorithm for each metric. These results indicate that the proposed algorithm is effective and robust for constrained multi-objective UAV path planning in complex static 3D environments.

## 1. Introduction

With ongoing advances in perception, decision-making, and environmental adaptability, unmanned autonomous systems have undergone increasingly diverse and intelligent development across multiple domains, such as unmanned aerial vehicles (UAVs) [1,2,3], intelligent robots [4,5], and autonomous vehicles [6]. Although these systems differ in their application scenarios, path planning, as a core technology for autonomous decision-making and safe navigation, is widely shared across different platforms and has become a major research topic in intelligent systems.

Depending on the operating environment, path planning for unmanned autonomous systems is typically classified into two categories: static and dynamic. Dynamic path planning [7] leverages online perception to make real-time adjustments subject to kinematic and dynamic constraints; however, its reliance on local sensory data often limits global path quality in complex environments. In contrast, static planning methods, such as the A* algorithm [8] and simulated annealing [9], rely on pre-modeled environments to obtain globally informed path solutions, making them well suited to medium- and long-range navigation tasks. Accordingly, this study focuses on static offline planning, where terrain information, obstacle distributions, and threat regions are assumed to be available before mission execution. This setting is particularly relevant to pre-mission global route generation in mapped environments. Unlike dynamic replanning, which requires online perception, state estimation, moving-obstacle prediction, and real-time trajectory updates, the present work aims to obtain globally informed Pareto trade-off paths in a pre-modeled environment. Nevertheless, traditional methods [10] often struggle to balance search efficiency, global optimality, and scalability in the presence of multiple objectives and complex constraints.

In UAV path planning tasks, the flight process must simultaneously satisfy both environmental and performance constraints [11]. Environmental constraints mainly arise from threat regions created by complex terrain and dense obstacles, whereas performance constraints relate to motion limitations such as flight altitude, turning angles, and trajectory smoothness. Consequently, UAV path planning aims to generate high-quality flight trajectories without violating these constraints [12], and can therefore be formulated as a constrained nonlinear multi-objective optimization problem. Under such a formulation, selecting an appropriate path planning algorithm is essential for achieving high-quality and stable flight trajectories.

In recent years, swarm intelligence (SI) optimization algorithms have been widely applied to UAV path planning problems [13]. By simulating cooperative behaviors and evolutionary mechanisms observed in natural populations, these algorithms establish heuristic search strategies with strong robustness and self-organizing learning capabilities, making them well-suited for solving complex nonlinear optimization problems. SI algorithms such as the Particle Swarm Optimization (PSO) [14], the Ant Colony Optimization (ACO) [15], the Gray Wolf Optimizer (GWO) [16], the Wild Horse Optimizer (WHO) [17], and the Coati Optimization Algorithm (COA) [18], together with their improved variants, have been successfully applied to UAV path planning and have demonstrated promising search performance in complex environments. Traditional single-objective optimization methods typically combine multiple performance indicators into a single objective using predefined weight coefficients. However, varying weight settings may yield different solutions, making it difficult to ensure solution stability. In contrast, multi-objective optimization algorithms can simultaneously handle multiple conflicting objectives without requiring predefined weights. By generating a set of non-dominated paths, they provide decision-makers with multiple feasible trade-off solutions, thereby offering greater flexibility and practical value in complex 3D UAV path planning problems [19].

### 1.1. Related Work

Existing UAV path planning methods can be broadly divided into classical planning methods, swarm-intelligence-based methods, and multi-objective optimization methods. Classical methods, such as A* and simulated annealing, are effective for structured or pre-modeled environments because of their clear search logic and relatively simple implementation. However, when the planning space becomes high-dimensional and involves multiple constraints, these methods may suffer from limited scalability, sensitivity to environment discretization, or difficulty in balancing multiple conflicting objectives.

Swarm intelligence optimization algorithms have been widely investigated for UAV path planning because of their population-based search ability and robustness in nonlinear optimization problems. Representative algorithms, including PSO, ACO, GWO, WHO, and COA, have shown promising performance in complex path planning tasks. Nevertheless, many SI-based UAV path planning studies are still formulated as single-objective or weighted-sum optimization problems. In such cases, the final solution may depend strongly on predefined weight coefficients, and it is difficult to provide a diverse set of Pareto trade-off paths for decision-making.

Multi-objective optimization methods provide a more suitable framework for UAV path planning because they can simultaneously optimize conflicting objectives without aggregating them into a single scalar function. Algorithms such as NSGA-II, MOPSO, and MOWOA can generate non-dominated solution sets and provide multiple candidate paths with different trade-offs. Among them, MOWOA extends WOA to multi-objective optimization by incorporating Pareto dominance and external archive maintenance. Its simple structure and few control parameters make it suitable for continuous multi-objective engineering optimization.

In addition to evolutionary and swarm-intelligence-based multi-objective optimizers, graph-based and fuzzy-logic-assisted multi-objective path planning methods have also been investigated in unmanned vehicle navigation. For example, SIGPA and its fuzzy-logic extension SIGPAF were developed for multi-objective route/path planning, while ACO-based comparative studies and fuzzy Pareto-optimality approaches further examined trade-offs among distance, safety, smoothness, and energy-related criteria in unmanned surface vehicle path planning [20,21,22,23]. These studies provide useful references for multi-objective path generation under conflicting criteria, although the present work focuses on constrained static 3D UAV path planning using a Pareto-based swarm intelligence optimizer.

Mirjalili et al. [24] proposed the whale optimization algorithm (WOA), which was inspired by the spiral bubble-net feeding behavior of humpback whales. Owing to its simple structure, few control parameters, and strong ability to escape local optima, WOA has demonstrated competitive performance in continuous optimization tasks. However, WOA is inherently a single-objective optimization algorithm, which limits its direct application to engineering problems involving multiple conflicting objectives [25]. To address this limitation, Huang et al. [26] introduced a multi-objective framework into WOA (MOWOA) for computation offloading in mobile edge computing. By incorporating non-dominated sorting and an external archive, MOWOA exploits Pareto dominance relationships to enable simultaneous multi-objective optimization. Experimental results demonstrated that MOWOA achieves favorable trade-offs among cost, energy, and latency, highlighting its potential in multi-objective engineering tasks. In addition to UAV-related optimization tasks, WOA has also been widely used as an important comparative metaheuristic in engineering optimization problems. In the field of large-scale structural damage detection, Ghannadi and Kourehli [27] investigated the recently developed optimization algorithms and compared their performance with several well-known metaheuristics, including WOA, ALO, and GOA. This comparison suggests that WOA remains a relevant benchmark in engineering optimization, while also indicating that problem-specific algorithmic improvements are often necessary when the optimization landscape becomes highly constrained or computationally demanding. Building upon this foundation, subsequent studies have sought to refine the convergence, diversity, and adaptability of MOWOA. Dwivedi et al. [28] proposed a refined version (RMOWOA) to enhance search efficiency and improve the uniformity of solution distribution in wireless sensor networks. Wang et al. [29] developed a Laplacian-enhanced MOWOA (LE-MOWOA), introducing a specialized search operator to improve global exploration and Pareto-front approximation. In addition, Ramos et al. [30] investigated MOWOA from a decomposition-based perspective (MOWOA/D), transforming multi-objective problems into scalar subproblems to extend the framework’s applicability.

MOWOA features a simple structure and ease of implementation and has achieved promising performance in a variety of multi-objective optimization problems. Its search strategy also provides a viable approach to UAV path planning. However, existing WOA/MOWOA variants are still less explored in constrained static 3D UAV path planning, where narrow feasible corridors, dense obstacles, and multiple conflicting objectives impose higher requirements on feasible-solution generation, search guidance, and Pareto-solution distribution [31]. Different from general hybrid WOA/MOWOA variants that mainly improve convergence, diversity, or decomposition strategies at the operator level, constrained 3D UAV path planning requires coordinated improvement across feasible-solution generation, leader-guided search, and archive distribution maintenance.

Despite these advances, several limitations remain when MOWOA and related multi-objective swarm intelligence algorithms are applied to constrained static 3D UAV path planning. First, random initialization may generate many infeasible paths in obstacle-dense environments, reducing the number of effective search samples. Second, single-leader guidance may bias the population toward limited compromise regions and weaken Pareto-front coverage. Third, conventional archive maintenance strategies may not sufficiently regulate the global distribution of non-dominated solutions under strong objective conflicts. To provide a clearer overview of the methodological background, Table 1 summarizes representative path planning and multi-objective optimization methods in terms of their categories, main advantages, and main limitations.

### 1.2. Motivation and Contributions

Based on the above review, this study focuses on offline multi-objective UAV path planning in constrained static 3D environments. The main challenge lies not only in optimizing several conflicting objectives but also in maintaining feasible-solution generation, stable search guidance, and well-distributed Pareto solutions under complex environmental constraints.

Recent studies on improved multi-objective swarm-intelligence algorithms suggest that effective algorithmic improvement is often achieved by addressing specific limitations of the original optimizer. Sun et al. [34] introduced an external archive-guided mechanism into a dynamic niching particle swarm optimizer, while Wang et al. [35] redesigned global-best selection and position updating in an adaptive distance-based multi-objective particle swarm optimizer. In multi-objective UAV path planning, Zhang et al. [36] incorporated multi-mode collaboration and reinforcement-learning-based mode selection into MOPSO to coordinate convergence and diversity. These studies indicate that problem-specific reconstruction of key search mechanisms is a meaningful strategy for complex multi-objective optimization tasks.

Although existing MOWOA-based methods provide a useful Pareto optimization framework, several mechanisms may become less effective in constrained 3D UAV path planning. Random initialization may generate many infeasible paths in environments with dense obstacles and narrow feasible corridors. Single-leader guidance may bias the population toward one local feasible region, thereby weakening the exploration of alternative trade-off solutions. Conventional archive maintenance mainly preserves nondominated solutions, but its distribution information is not sufficiently used for leader selection and search feedback.

To address these limitations, this study proposes an Improved Multi-Leader Multi-Objective Whale Optimization Algorithm (IML-MOWOA). Rather than treating the proposed strategies as independent add-on operators, IML-MOWOA reconstructs the search-information flow of MOWOA at three coupled levels: feasibility-oriented candidate generation, density-aware archive regulation, and multi-leader feedback updating. The main contributions are summarized as follows:

1. An adaptive opposition-based learning (AOBL) strategy is designed to improve the feasibility and spatial coverage of the initial population, thereby providing higher-quality candidates for subsequent archive updating and search guidance.
2. A multi-leader dynamic weighting mechanism with adaptive cosine annealing is proposed to alleviate single-leader search bias. By replacing the original single-leader guidance term with a dynamically weighted multi-leader attractor in the position-update process, the method strengthens multi-directional search capability.
3. A grid-based external archive update mechanism is designed to improve Pareto-solution distribution. The grid-density information is further used to provide density-aware leader-selection information, thereby coupling archive maintenance with search guidance.

Simulation experiments in multiple static 3D scenarios indicate that IML-MOWOA achieves competitive overall performance, with improved convergence behavior, Pareto-front distribution, feasible-path generation, and task-level path quality within the considered offline static planning setting.

## 2. Problem Description

### 2.1. Multi-Objective Optimization Problem

Multi-objective optimization problems (MOPs) involve multiple mutually conflicting objective functions. Considering a minimization problem, the decision vector is denoted by $X\in \Omega \subset R^{D}$, where *D* denotes the dimension of the decision variables and Ω represents the feasible region. Let *V* denote the number of objective functions. The corresponding MOP can be expressed as:

(1) $$\min_{X\in \Omega }\;F(X)=(f_{1}(X),f_{2}(X),\ldots ,f_{V}(X))$$

Because of the inherent conflicts among objectives, a single solution that simultaneously optimizes all objectives usually does not exist. Instead, a multi-objective optimization problem generally yields a set of Pareto-optimal trade-off solutions, whose image in the objective space forms the Pareto front [16].

### 2.2. UAV Path Planning

The UAV path planning problem involves multiple optimization objectives [11], including path length $f_{1}$, threat cost $f_{2}$, smoothness cost $f_{3}$, and altitude cost $f_{4}$. To achieve an optimal balance among these competing requirements, these criteria are formulated as individual objective functions within a multi-objective optimization framework. Consequently, the UAV path planning task is defined as a constrained multi-objective optimization problem.

#### 2.2.1. Path Length

In various environments, finding the shortest path from the start point to the end point without colliding with obstacles has become an important criterion for evaluating algorithm performance. Therefore, the path length is defined as the first objective function $f_{1}$. Assume that a path contains *k* waypoints, including the start and end points, and the *i*-th path is represented as $X_{i}=\left(w_{i,1},w_{i,2},\ldots ,w_{i,k}\right)$, where the *j*-th waypoint of the *i*-th path can be expressed by coordinates $w_{i,j}=(x_{i,j},y_{i,j},z_{i,j})$. Since the path $X_{i}$ consists of *k* path points, including the start point and the end point, the total path length is calculated by summing the Euclidean lengths of the *k* − 1 consecutive path segments. The path length objective function is defined as follows:

(2) $$f_{1}(X_{i})={\sum_{j=1}^{k-1}\left\|w_{i,j+1}-w_{i,j}\right\|}_{2}$$

This objective is derived from the Euclidean geometry of consecutive waypoint segments and measures the total flight distance of the generated trajectory. Minimizing $f_{1}$ encourages the algorithm to search for shorter and more efficient paths, and it can be regarded as a geometric proxy for flight efficiency within the offline path planning framework.

#### 2.2.2. Threat Cost

**Figure 1:**
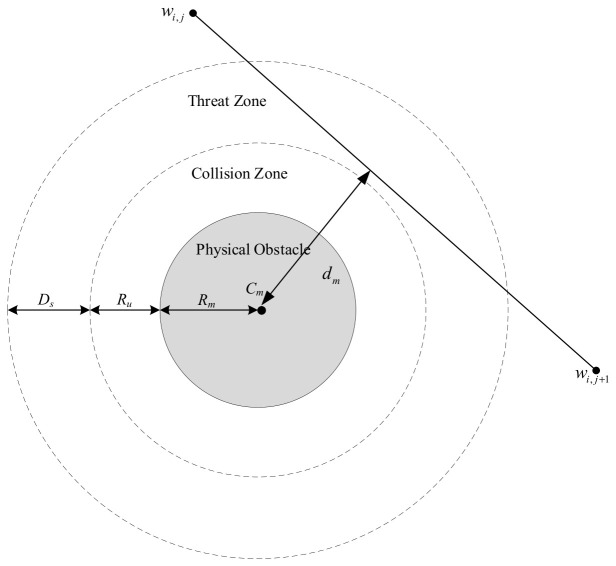
Top-view geometry of the obstacle, collision zone, and threat zone.

Because the UAV flight path must avoid all obstacles, the threat cost is defined as the second objective function, $f_{2}$. To facilitate threat cost evaluation, each obstacle is approximated as a vertical cylinder whose base is anchored on the local terrain surface, and its height is defined as the vertical distance from the terrain surface to the top surface of the cylinder. Let M denote the set of all obstacles, let $C_{m}$ denote the center of the horizontal projection of the *m*-th obstacle, let $R_{m}$ denote its radius, and let $D_{s}$ denote the buffer-zone size. $R_{u}$ represents the safety radius of the UAV and $d_{m}$ denotes the minimum Euclidean distance from the path segment $\vec{w_{i,j}w_{i,j+1}}$ to the obstacle center $C_{m}$. The top-view geometry of the obstacle model is illustrated in Figure 1.

For the obstacle set M, the threat cost $f_{2}$ of the path $X_{i}$ is given by Equation (3).

(3) $$f_{2}(X_{i})=\sum_{j=1}^{k-1}\sum_{m=1}^{\left|M\right|}T_{m}(\vec{w_{i,j}w_{i,j+1}})$$

(4) $$T_{m}(\bar{w_{i,j}w_{i,j+1}})=\left\{\begin{array}{cc} \infty & d_{m}<R_{m}+R_{u}\\ (R_{m}+R_{u}+D_{s})-d_{m} & R_{m}+R_{u}\leq d_{m}<R_{m}+R_{u}+D_{s}\\ 0 & d_{m}\geq R_{m}+R_{u}+D_{s} \end{array}\right.$$

This objective is derived from the geometric relationship between each path segment and the cylindrical obstacle-threat region. The physical obstacle region represents direct collision risk, whereas the surrounding buffer region reflects potential threat exposure; therefore, minimizing $f_{2}$ encourages the generated path to maintain sufficient clearance from obstacles and threat regions.

#### 2.2.3. Smoothness Cost

**Figure 2:**
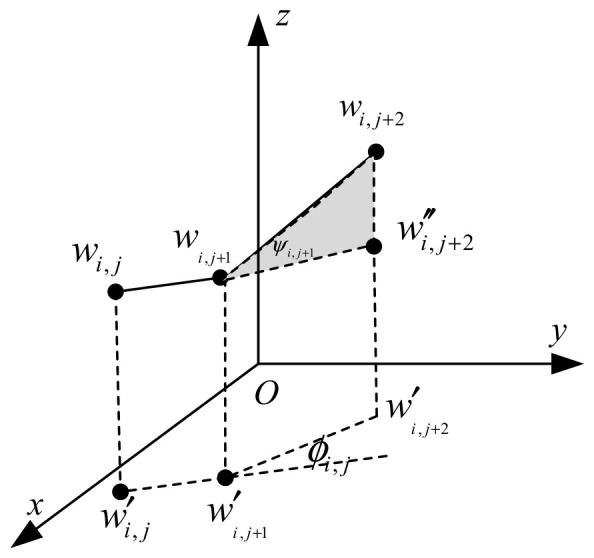
The turning angle and climbing angle.

The smoothness of the flight path is mainly affected by two geometric angles, namely the turning angle and the climbing angle. Accordingly, the smoothness cost characterized by these two angles is defined as the third objective function, $f_{3}$. Figure 2 illustrates the local geometric relationship among three consecutive waypoints, $w_{i,j}$, $w_{i,j+1}$, and $w_{i,j+2}$ in a 3D coordinate system.

The points ${w^{\prime }}_{i,j}$, ${w^{\prime }}_{i,j+1}$, and ${w^{\prime }}_{i,j+2}$ represent the projections of $w_{i,j}$, $w_{i,j+1}$, and $w_{i,j+2}$ onto the *xy*-plane, respectively. After projecting the consecutive path segments $\vec{w_{i,j}w_{i,j+1}}$ and $\vec{w_{i,j+1}w_{i,j+2}}$ onto the *xy*-plane, the angle between the extension of $\vec{{w^{\prime }}_{i,j}{w^{\prime }}_{i,j+1}}$ and the path segment $\vec{{w^{\prime }}_{i,j+1}{w^{\prime }}_{i,j+2}}$ is defined as the turning angle $\varphi_{i,j}$, as given in Equation (5):

(5) $$\varphi_{i,j}=\arctan(\frac{\left\|\vec{{w^{\prime }}_{i,j}{w^{\prime }}_{i,j+1}}\times \vec{{w^{\prime }}_{i,j+1}{w^{\prime }}_{i,j+2}}\right\|}{\vec{{w^{\prime }}_{i,j}{w^{\prime }}_{i,j+1}}\cdot \vec{{w^{\prime }}_{i,j+1}{w^{\prime }}_{i,j+2}}})$$

${w^{\prime \prime }}_{i,j+1}$ denotes the projection of the waypoint $w_{i,j+1}$ onto the vertical plane passing through the segment $\vec{w_{i,j}w_{i,j+1}}$. The angle formed between the path segment $\vec{w_{i,j}w_{i,j+1}}$ and its projection $\vec{w_{i,j}{w^{\prime \prime }}_{i,j+1}}$ on the corresponding vertical plane is defined as the climbing angle $\psi_{i,j}$. The calculation formula of $\psi_{i,j}$ is given in Equation (6):

(6) $$\psi_{i,j}=\arctan(\frac{z_{i,j+1}-z_{i,j}}{\left\|\vec{{w^{\prime }}_{i,j}{w^{\prime }}_{i,j+1}}\right\|})$$

where $z_{i,j+1}$ and $z_{i,j}$ represent the coordinate values of the *j* + 1-th waypoint on the *z*-axis.

The turning angle $\varphi_{i,j}$ is used to measure path smoothness in the horizontal direction, whereas the climbing angle $\psi_{i,j}$ characterizes variation in the vertical direction. Therefore, the mathematical formulation of the smoothness cost $f_{3}$ is defined as follows:

(7) $$f_{3}(X_{i})=\sum_{j=1}^{k-2}\varphi_{i,j}+\sum_{j=1}^{k-1}\psi_{i,j}$$

This objective is derived from the turning and climbing angles formed by consecutive path segments, which characterize the horizontal and vertical maneuvering intensity of the UAV. Minimizing $f_{3}$ reduces abrupt heading changes and steep climbing or descending maneuvers, thereby improving trajectory continuity and flight feasibility.

#### 2.2.4. Altitude Cost

During UAV operation, selecting an appropriate flight altitude is crucial. In this study, the UAV flight altitude is constrained within a specified range. The altitude cost penalizes both altitude violations and deviations from the preferred altitude band center. The mathematical formulation of the altitude cost $f_{4}$ is given as follows:

(8) $$f_{4}(X_{i})=\sum_{j=1}^{k}H_{i,j}$$

(9) $$H_{i,j}=\left\{\begin{array}{cc} \left|h_{i,j}-\frac{1}{2}(h_{\max}+h_{\min})\right| & h_{\min}\leq h_{i,j}\leq h_{\max}\\ \infty & \mathrm{otherwise} \end{array}\right.$$

where $h_{i,j}$ refer to the altitude of the *j*-th waypoint in the *i*-th path, with $h_{\max}$ and $h_{\min}$ representing the maximum and minimum allowable flight altitudes, respectively.

The altitude-cost formulation reflects the allowable flight-altitude envelope and the preference for maintaining a stable altitude within the feasible band. Minimizing $f_{4}$ helps maintain altitude safety and vertical stability, while preventing the algorithm from selecting paths with unsafe or inefficient altitude profiles.

In the numerical implementation, the infinite penalties in the threat-cost and altitude-cost formulations are used only to indicate hard constraint violations, and non-finite objective values are replaced by a large finite penalty value before sorting, archive updating, and statistical calculation.

## 3. Multi-Objective Whale Optimization Algorithm

### 3.1. Principles of the Whale Optimization Algorithm

Since the UAV path planning problem considered in this study is encoded as a continuous waypoint-based decision vector, it is suitable for population-based continuous optimization. WOA provides a simple and parameter-efficient search framework with global exploration and local exploitation capabilities through random search, encircling behavior, and spiral updating. Its multi-objective extension, MOWOA, further incorporates Pareto dominance and external archive maintenance, making it compatible with the multi-objective formulation of UAV path planning. However, conventional MOWOA still faces limitations in constrained static 3D environments, including a low feasible-solution ratio, single-leader search bias, and insufficient Pareto-solution distribution control. Therefore, MOWOA is adopted as the base framework and further improved through feasibility-oriented initialization, multi-leader dynamic guidance, and grid-based archive maintenance.

WOA is an SI algorithm inspired by the bubble-net feeding strategy of humpback whales. This cooperative hunting behavior is mainly characterized by three mechanisms, i.e., encircling prey, bubble-net attacking, and random search, which enable the algorithm to balance exploration and exploitation in the search space.

In the optimization process, the position of the *i*-th whale at iteration *t* is represented by a position vector $X_{i}(t)$. For the UAV path planning problem considered in this study, the position vector corresponds to the waypoint variables of a candidate trajectory and can be written as:

(10) $$X_{i}(t)={\left[w_{i,j}(t)\right]}_{j=1}^{k}$$

where *k* denotes the total number of waypoints and $w_{i,j}(t)$ represents the waypoint variable associated with the *j*-th intermediate waypoint of the *i*-th candidate path.

#### 3.1.1. Encircling Prey

In the encircling mechanism, whales update their positions toward the current best solution. The mathematical model is expressed as follows:

(11) $$X(t+1)=X^{*}(t)-A\cdot D$$

(12) $$D=\left|C\cdot X^{*}(t)-X(t)\right|$$

where $X^{*}(t)$ denotes the best position in the population, and *t* is the current iteration number. The coefficient vectors A and C are defined as follows:

(13) $$A=2a\cdot r_{1}-a,\;C=2\cdot r_{2},\;a=2-\frac{2t}{T_{\max}}$$

where a decreases linearly from 2 to 0 during the iterations, $r_{1}$ and $r_{2}$ are random values uniformly distributed in $\left[0,1\right]$, and $T_{\max}$ is the maximum number of iterations.

#### 3.1.2. Bubble-Net Attack Mechanism

When whales approach the prey, they follow a logarithmic spiral trajectory toward the current best position. The distance between the whale and the current best position is defined as:

(14) $$D^{s}=\left|X^{*}(t)-X(t)\right|$$

The spiral updating rule is given by:

(15) $$X(t+1)=D^{s}\cdot e^{bl}\cdot \cos(2\pi l)+X^{*}(t)$$

where $D^{s}$ denotes the Euclidean distance between the current individual and the current best solution, b controls the spiral shape, and $l\in \left[-1,1\right]$ is a random number.

The encircling and spiral updating mechanisms are selected with equal probability. Accordingly, the position update rule can be expressed as:

(16) $$X(t+1)=\left\{\begin{array}{ll} \begin{array}{c} X^{*}(t)-A\cdot D \end{array} & p<0.5\\ \begin{array}{c} D^{s}\cdot e^{bl}\cdot \cos(2\pi l)+X^{*}(t) \end{array} & p\geq 0.5 \end{array}\right.$$

where $p\in \left[0,1\right]$ is a uniformly distributed random number.

#### 3.1.3. Random Search for Prey

To enhance exploration capability, WOA performs random search when $\left|A\right|\geq 1$. In this case, each whale randomly selects another individual from the population as a reference:

(17) $$D^{r}=\left|C\cdot X_{rand}(t)-X(t)\right|$$

(18) $$X(t+1)=X_{rand}(t)-A\cdot D^{r}$$

where $D^{r}$ represents the Euclidean distance between the current individual and a randomly selected one, and $X_{rand}(t)$ denotes the position vector of a randomly selected whale from the population. When $\left|A\right|<1$ the whale performs an encircling behavior toward the prey.

### 3.2. Principle of the Multi-Objective Whale Optimization Algorithm

Although WOA demonstrates strong optimization capability for single-objective problems, many real-world engineering applications involve multiple conflicting objectives. To address this limitation, MOWOA extends WOA to multi-objective optimization problems. Unlike WOA, which seeks a single optimal solution, MOWOA aims to obtain a set of trade-off solutions whose image in the objective space forms the Pareto front. During the optimization process, individuals are evaluated according to Pareto dominance relationships, and the resulting non-dominated solutions are stored in an external archive.

At each generation, the current population is merged with the archive, and non-dominated sorting is performed to dynamically update the archive. To prevent uncontrolled growth of the archive, its size is regulated using the crowding-distance strategy. When the number of archived solutions exceeds the predefined capacity, individuals located in densely populated regions of the objective space are preferentially removed, thereby maintaining a balanced distribution of solutions. Meanwhile, the archive also provides guidance information for the search process. Individuals located in sparsely populated regions are typically selected from the archive as global leaders to guide the population toward diverse regions of the Pareto front.

Although Pareto-based ranking avoids the limitations of aggregating multiple objectives into a single weighted fitness function, it does not explicitly capture local density information in the objective space. As a consequence, the diversity of the obtained solutions may deteriorate during the evolutionary process. In addition, the crowding-distance-based leader selection strategy may generate loosely distributed solutions that deviate from the true Pareto front, thereby limiting overall optimization performance in complex tasks such as UAV path planning.

## 4. Improved Multi-Objective Whale Optimization Algorithm

### 4.1. Adaptive Opposition-Based Learning Initialization

In multi-objective optimization algorithms, the population plays a crucial role in overall algorithm performance. It not only affects the exploration capability during the early search stage but also directly influences the proportion of feasible solutions and the quality of subsequent convergence. MOWOA employs uniform random sampling to generate the initial population, which can provide good spatial coverage in unconstrained or weakly constrained problems. However, complex 3D UAV path planning problems involve multiple constraints and performance requirements, such as threat avoidance, altitude limits, and trajectory smoothness requirements. Feasible solutions are often distributed within narrow and discrete safe regions, resulting in a highly constrained decision space with a very small feasible domain. Consequently, random initialization may generate a large number of infeasible paths, which reduces the proportion of effective samples, weakens early exploration capability, and may even lead to premature convergence.

To address these issues, this study introduces an Adaptive Opposition-Based Learning (AOBL) initialization strategy based on Opposition-Based Learning (OBL) theory. The proposed strategy enhances the coverage of the search space by jointly evaluating individuals and their corresponding opposite solutions.

Recent work has also shown that OBL-enhanced metaheuristics can improve population diversity and optimization performance in path planning problems. For example, Ntakolia [37] combined OBL with the Fick’s Law Algorithm and further developed a fuzzy OBL-based variant for UAV path planning with obstacle avoidance, demonstrating the usefulness of opposition-based population enhancement in improving solution quality and search efficiency. However, standard OBL commonly relies on globally defined opposition mappings. Unlike standard OBL, the AOBL strategy in this study utilizes population statistics to construct adaptive reflection intervals and updates the reflection scale using non-dominated sorting feedback. This mechanism allows opposite samples to better align with the distribution of the current solution set and shift toward potentially feasible regions, thereby improving the feasibility rate of initial paths while maintaining population diversity.

#### 4.1.1. Theoretical Foundation and Adaptive Reflection Mechanism

OBL, first proposed by Tizhoosh, generates an opposite solution for a given current solution, thereby increasing the probability of obtaining a better candidate. For the *d*-th component of a waypoint $w_{i,j}$, where $d\in \left\{x,y,z\right\}$, $lb_{d}$ and $ub_{d}$ denote the predefined global lower and upper search bounds of this coordinate dimension, respectively. These two terms are fixed boundary values determined by the search space rather than waypoint variables. Therefore, $w_{i,j}^{\left(d\right)}\in \left[lb_{d},ub_{d}\right]$, and according to OBL theory, the corresponding opposite component is defined as follows:

(19) $${\tilde{w}}_{i,j}^{\left(d\right)}=lb_{d}+ub_{d}-w_{i,j}^{\left(d\right)}$$

Under the assumption of a uniform distribution, the probability that opposite samples fall into feasible or promising regions is at least as high as that of the initial samples. Therefore, jointly evaluating the original and opposite solutions can effectively enhance search space coverage.

In UAV path planning problems, due to the presence of threat regions, terrain undulations, and various flight constraints, feasible paths are typically distributed within limited and discrete safe regions. When random initialization is used to generate the initial population, many candidate paths may fall into infeasible regions, resulting in a low proportion of feasible solutions in the initial population. The opposition-based learning strategy constructs opposite paths during candidate generation, thereby improving decision-space coverage and increasing the probability of obtaining feasible paths.

However, the OBL strategy relies on fixed global bounds to construct opposite samples. In complex UAV path planning problems, where feasible regions are sparse, irregular, and locally clustered, such global reflection may generate samples that are insufficiently aligned with the current population distribution. Therefore, to better adapt the reflection process to the local statistical characteristics of promising regions, an adaptive reflection mechanism is further introduced.

For the *d*-th coordinate component of the waypoint $w_{i,j}$, the mean and standard deviation of the current population are calculated as follows:

(20) $$\begin{array}{c} \mu_{j,d}=\frac{1}{N}\sum_{i=1}^{N}w_{i,j}^{\left(d\right)}\\ \sigma_{j,d}=\sqrt{\frac{1}{N-1}\sum_{i=1}^{N}{\left(w_{i,j}^{\left(d\right)}-\mu_{j,d}\right)}^{2}} \end{array}$$

where *N* denotes the population size. Based on the population statistical distribution, the local statistical interval can be constructed as follows:

(21) $$\begin{array}{c} l{b^{\prime }}_{j,d}=\mu_{j,d}-\partial \sigma_{j,d}\\ u{b^{\prime }}_{j,d}=\mu_{j,d}+\partial \sigma_{j,d} \end{array}$$

The interval $\left[l{b^{\prime }}_{j,d},u{b^{\prime }}_{j,d}\right]$ characterizes the local statistical reflection range around the current population center and provides the basis for adaptive scaling in the reflection process, where ∂ is the reflection scale factor used to adjust the exploration range. Taking $\mu_{j,d}$ as the reflection center and ∂ as the reflection intensity factor, the adaptive opposition mapping is performed within the local statistical interval $\left[l{b^{\prime }}_{j,d},u{b^{\prime }}_{j,d}\right]$ and is defined as follows:

(22) $${\tilde{w}}_{i,j}^{\left(d\right)}=\min(u{b^{\prime }}_{j,d},\max(l{b^{\prime }}_{j,d},\mu_{j,d}+\partial (\mu_{j,d}-w_{i,j}^{\left(d\right)})))$$

Unlike the traditional fixed-boundary reflection mechanism, this method performs adaptive mapping centered on the current population statistics. As a result, newly generated samples tend to be distributed in underexplored complementary regions of the search space, thereby improving initialization quality while maintaining population diversity. This mechanism implements an adaptive reflection strategy guided by the statistical distribution of the current population, thereby providing higher-quality candidate paths for subsequent optimization.

#### 4.1.2. Reflection Scale Factor Adjustment via Performance Feedback

In multi-objective optimization problems, the initialization stage should avoid using weighted or fixed scalarization schemes to evaluate individual quality, as this may introduce implicit single-objective bias and compromise Pareto consistency. Therefore, a performance comparison mechanism is established based on non-dominated sorting to evaluate the overall performance of the opposite sample set relative to the original sample set and to adaptively update the reflection scale factor accordingly.

First, the initial sample set X and the opposite sample set $\tilde{X}$ are merged to form a candidate set:

(23) $$U=X\cup \tilde{X}$$

Non-dominated sorting is then performed on the set U to obtain the first non-dominated front $F_{1}(U)$. Decision vectors in this front are mutually non-dominated in the Pareto sense and represent the best trade-off solutions within the current candidate set.

To characterize the relative contributions of the original and opposite samples in the first non-dominated front, the proportions of opposite and original decision vectors in the first non-dominated front are defined as follows:

(24) $$\begin{array}{c} r_{\tilde{X}}=\frac{\left|F_{1}(U)\cap \tilde{X}\right|}{\left|F_{1}(U)\right|}\\ r_{X}=\frac{\left|F_{1}(U)\cap X\right|}{\left|F_{1}(U)\right|} \end{array}$$

If $r_{\tilde{X}}>r_{X}$, it indicates that opposite samples account for a larger proportion in the first non-dominated front, suggesting that their overall multi-objective performance is superior to that of the original samples. Otherwise, the opposite samples are considered not to offer significant advantages. Based on this observation, the performance feedback signal ΔQ is defined as follows:

(25) $$\Delta Q=\left\{\begin{array}{ll} 1 & r_{\tilde{X}}>r_{X}\\ 0 & r_{\tilde{X}}=r_{X}\\ -1 & r_{\tilde{X}}<r_{X} \end{array}\right.$$

when ΔQ=1, the opposite samples generated by the current reflection mapping exhibit relatively high overall quality, and the reflection range is expanded to enhance global exploration capability. When ΔQ=0, the performance of the two types of samples in the first non-dominated front is considered comparable, and the reflection scale remains unchanged. When ΔQ=−1, the reflection scale is reduced to maintain search stability.

Accordingly, the adaptive update strategy for the reflection scale factor ∂ is defined as follows:

(26) $$\partial_{new}=\left\{\begin{array}{ll} \min(\partial +\gamma ,\partial_{\max}) & \Delta Q=1\\ \partial & \Delta Q=0\\ \max(\partial -\gamma ,\partial_{\min}) & \Delta Q=-1 \end{array}\right.$$

where γ denotes the adjustment step size, while $\partial_{\min}$ and $\partial_{\max}$ represent the lower and upper bounds of the reflection scale, respectively. To prevent excessive oscillation during the initialization stage, the step size γ is set to control the adjustment magnitude of the reflection scale, thereby balancing exploration and stability during initialization and facilitating a gradual transition from exploration to convergence.

#### 4.1.3. Initialization Procedure

The AOBL initialization procedure is summarized as follows:

Step 1: Random sampling. Generate the initial sample set X within the global bounds.

Step 2: Statistical analysis. Compute the population means and standard deviation for each dimension.

Step 3: Adaptive opposition mapping. Generate the opposite sample set $\tilde{X}$.

Step 4: Candidate merging. Combine X and $\tilde{X}$ to form the candidate sample set U.

Step 5: Performance evaluation. Compute the performance feedback signal based on the non-dominated sorting results, and update the reflection scale factor for subsequent adaptive adjustment. Then, repeat Steps 2–4.

Step 6: Population screening. Select the adaptively optimized initial population $P_{0}$.

### 4.2. Dynamic Weighted Multi-Leader Guidance Mechanism

Although the AOBL strategy improves the quality of the initial population, effective search guidance during the iterative optimization process remains critical for obtaining a well-distributed Pareto solution set. Therefore, this section introduces a dynamic weighted multi-leader guidance mechanism.

In multi-objective path planning models, conflicts inherently exist among different objective functions, and the resulting trade-off solutions are distributed along the Pareto front. Therefore, no single individual can simultaneously represent all trade-off directions. In the multi-objective whale optimization framework, non-dominated sorting and an external archive are used to store non-dominated solutions, thereby approximating the Pareto front while maintaining distribution diversity. However, WOA and its multi-objective variants typically adopt a single leader to guide population updates. This single-leader guidance mechanism may cause the population to converge toward local compromise regions, thereby limiting the coverage of the Pareto front and reducing solution diversity.

To address this limitation, this paper proposes a multi-leader dynamic weighted guidance mechanism. In this approach, multiple representative non-dominated solutions are selected from the external archive, their corresponding decision vectors are used as leaders, and their guidance directions are integrated through adaptive weight fusion. As a result, multi-directional collaborative search is achieved while enabling stage-wise convergence control.

#### 4.2.1. Multi-Leader Selection Model

To enhance the representativeness of decision vectors in the objective space and improve Pareto-front coverage, this study adopts a sparsity-prioritized selection strategy based on grid density. Specifically, several representative non-dominated solutions located in low-density regions are selected from the external archive as leaders. Because too few decision vectors cannot adequately characterize the multi-directional features of the Pareto front, whereas too many introduce redundant information and increase update instability, three directionally complementary decision vectors are selected to form the leader set, thereby ensuring directional diversity while maintaining computational efficiency.

(27) $$X_{Leader}=\left\{X_{\alpha },X_{\beta },X_{\delta }\right\}$$

By prioritizing the selection of non-dominated solutions located in sparse regions, the population is guided toward underexplored regions of the Pareto front, thereby improving the uniformity of the solution distribution and suppressing local aggregation. To integrate the guidance information from multiple decision vectors, a multi-leader fusion model is constructed. The decision vectors in the leader set are combined to form a comprehensive decision vector:

(28) $$X_{L}(t)=\sum_{q\in \left\{\alpha ,\beta ,\delta \right\}}\omega_{q}(t)X_{q}(t)$$

where $\omega_{q}(t)$ denotes the dynamic weight of the leader vector indexed by q and satisfies the normalization constraints in Equation (29).

(29) $$\begin{array}{cc} \sum_{q\in \left\{\alpha ,\beta ,\delta \right\}}\omega_{q}(t)=1 & \omega_{q}(t)\geq 0 \end{array}$$

The comprehensive decision vector $X_{L}(t)$ replaces the single-leader position $X^{*}(t)$. This fusion strategy preserves information from multiple Pareto trade-off directions, enabling multi-directional collaborative guidance while improving the stability of the search process and maintaining the distribution quality of the Pareto solution set.

More specifically, the proposed multi-leader guidance mechanism can be interpreted as a density-aware search guidance strategy for maintaining Pareto-front diversity. In conventional MOWOA, population updating is guided by a single archived solution, which may cause individuals to move toward a limited compromise region. In contrast, the proposed mechanism selects representative leaders from sparsely populated regions of the external archive and integrates their decision vectors through dynamic Softmax weighting. As a result, multiple trade-off directions can participate in the position-update process, thereby reducing single-leader search bias and providing more balanced guidance toward different regions of the Pareto front.

#### 4.2.2. Softmax-Based Dynamic Weighting Strategy

Fixed weights cannot capture the stage-dependent differences in the contributions of different leaders during the search process, which may easily lead to long-term bias in the guidance direction and consequently weaken the adaptive capability of the algorithm. To enable dynamic adjustment of leader influence, this study develops a performance-based weight allocation model. The multi-objective performance of the leader indexed by q is evaluated using weighted Chebyshev scalarization:

(30) $$\begin{array}{cc} s_{q}=\underset{m}{\max}\left\{\lambda_{m}|f_{m}(X_{q})-r_{m}^{*}|\right\} & q\in \left\{\alpha ,\beta ,\delta \right\} \end{array}$$

where $\lambda_{m}$ is the weight coefficient, $f_{m}(\cdot )$ is the *m*-th objective function, and $r_{m}^{*}$ denotes the best objective value of the *m*-th objective among the current three leaders, which is used as the reference value for performance evaluation. The Softmax function is then employed to transform the scalarization results into dynamic weights:

(31) $$\omega_{q}(t)=\frac{e^{-\eta (t)(s_{q}-s_{\min})}}{\sum_{j\in \left\{\alpha ,\beta ,\delta \right\}}e^{-\eta (t)(s_{j}-s_{\min})}}$$

where $s_{q}$ denotes the scalarized performance metric of the *q*-th decision vector, $s_{\min}$ is the minimum evaluation score in the current leader set, and η(t) is a temperature parameter dynamically adjusted according to the iteration count. This parameter determines the distribution shape of the Softmax function and thus controls the relative guidance intensity of each leader. Since fixed parameters cannot simultaneously satisfy the exploration requirements of the early stage and the convergence requirements of the later stage, this paper introduces a staged temperature control strategy. The temperature parameter is defined as:

(32) $$\eta (t)=\eta_{\min}+(\eta_{\max}-\eta_{\min})\cdot g(t)$$

where $\eta_{\min}$ and $\eta_{\max}$ denote the lower and upper bounds of the temperature parameter, respectively. The temperature scheduling function g(t) is defined as a piecewise cosine form:

(33) $$g(t)=\left\{\begin{array}{cc} 0 & 0\leq t\leq \theta T_{\max}\\ \frac{1-\cos(\pi (\frac{t-\theta T_{\max}}{(1-\theta )T_{\max}}))}{2} & \theta T_{\max}<t\leq T_{\max} \end{array}\right.$$

where $\theta \in \left[0,1\right)$ represents the proportion of the initial constant-temperature phase and is used to control the duration of the early balanced search stage. In this study, θ=0.3 is adopted so that the algorithm maintains balanced multi-leader guidance during approximately the first 30% of the iterations, thereby promoting sufficient coverage of the objective space and enhancing global exploration capability. The sensitivity analysis reported in Section 5.2.6 shows that θ=0.3 achieves the lowest mean IGD across the three scenarios among the tested values, indicating that this setting provides a favorable balance between early multi-leader exploration and later exploitative guidance.

Through Softmax-based normalization, the weights of decision vectors in the leader set vary adaptively with their performance, such that better-performing decision vectors receive larger weights and exert stronger guidance. This weight allocation mechanism enables the search direction to adapt dynamically to the population state, thereby achieving a dynamic balance between multi-directional exploration and the exploitation of advantageous regions.

#### 4.2.3. Multi-Leader Dynamic Weighted Update Rule

Based on the aforementioned multi-leader fusion and dynamic weight allocation mechanism, the position update strategy of WOA is correspondingly extended. The composite leader vector $X_{L}(t)$ replaces the single-leader guidance as the search guidance center and participates in both the encircling and spiral update processes.

In the encircling phase, the distance between an individual and the comprehensive decision vector $D_{L}$ is defined as:

(34) $$D_{L}=\left|C\cdot X_{L}(t)-X_{i}(t)\right|$$

and the position is updated based on the comprehensive guidance distance:

(35) $$X_{i}(t+1)=X_{L}(t)-A\cdot D_{L}$$

where the expressions for the coefficient vectors A and C are calculated according to Equation (13).

In the spiral search phase, the comprehensive decision vector $D_{L}^{s}$ is introduced as the rotation center:

(36) $$D_{L}^{s}=\left|X_{L}(t)-X_{i}(t)\right|$$

(37) $$X_{i}(t+1)=D_{L}^{s}\cdot e^{bl}\cdot \cos(2\pi l)+X_{L}(t)$$

The above update rules preserve the original search framework of WOA while extending single-leader guidance to multi-leader collaborative guidance, enabling decision vectors to be simultaneously guided by information from multiple Pareto trade-off directions. As a result, search stability is enhanced, and Pareto-front coverage is improved.

### 4.3. Grid-Based External Archive Update Mechanism

MOWOA employs the crowding-distance mechanism to maintain the diversity of the external archive. However, this method primarily relies on local neighborhood information and cannot effectively capture the global distribution of solutions in the objective space. Moreover, as the archive size increases, the computational cost of maintaining the archive also increases. For problems with strongly conflicting objectives, such as 3D UAV path planning, relying solely on crowding distance makes it difficult to simultaneously maintain distribution uniformity and achieve management efficiency.

To address these limitations, this paper introduces a grid-based external archive update mechanism. In this approach, the objective space is discretized into regular grids, and both archive truncation and leader selection are performed based on grid density. This strategy improves the distribution uniformity of the Pareto solution set and enhances search stability.

#### 4.3.1. Objective Space Normalization and Grid Mapping

The external archive maintains diversity among non-dominated solutions using the crowding-distance metric. However, crowding distance relies on Euclidean distances in the objective space to estimate local density. When the number of objectives increases or the scales of different objectives differ significantly, this method may suffer from scale bias and high computational cost, which can reduce the efficiency and stability of archive maintenance. Therefore, it is necessary to construct a unified and globally consistent density representation to replace distance-based metrics.

To this end, the non-dominated solutions in the temporary archive are first normalized to eliminate the influence of different objective scales. There are *V* objectives, and the normalized value of the *v*-th objective is defined as:

(38) $$\begin{array}{cc} f_{v}^{norm}=\left\{\begin{array}{cc} \frac{f_{v}-f_{v}^{\min}}{f_{v}^{\max}-f_{v}^{\min}} & f_{v}^{\min}\neq f_{v}^{\max}\\ 0 & f_{v}^{\min}=f_{v}^{\max} \end{array}\right. & v=1,2,\ldots ,V \end{array}$$

where $f_{v}^{\min}$ and $f_{v}^{\max}$ respectively denote the minimum and maximum values of the *v*-th objective function in the current temporary archive.

Subsequently, each objective dimension is uniformly divided into $n_{grid}$ intervals. For any non-dominated solution, the grid index in the *v*-th objective dimension is defined as:

(39) $$\begin{array}{cc} c_{v}=\min(n_{grid},\left\lfloor f_{v}^{norm}n_{grid}\right\rfloor +1), & v=1,2,\ldots ,V \end{array}$$

Based on these indices, the multi-dimensional grid coordinates $\left(c_{1},c_{2},\ldots ,c_{V}\right)$ can be uniquely mapped to a one-dimensional grid identifier:

(40) $$G_{k}=1+\sum_{v=1}^{V}(c_{v}-1)n_{grid}^{v-1}$$

Through this mapping process, the continuous objective space is transformed into a discrete grid structure, which provides the basis for subsequent density estimation and archive update operations.

#### 4.3.2. Grid-Density-Driven Archive Update Mechanism

Let ρ(G) denote the number of solutions assigned to the grid G, which serves as the density measure for that region. In each iteration, the current population is first merged with the existing external archive, and the non-dominated solutions are extracted to form a temporary archive. Subsequently, the grid assignment of each archived solution is determined according to Equations (38) and (39), and the density of each grid is computed.

When the size of the temporary archive exceeds the upper limit $N_{r}$, solutions are preferentially removed from the densest grid to suppress excessive aggregation in local regions. The densest grid is defined as:

(41) $$G^{*}=\arg\underset{G}{\max}\;\rho (G)$$

If multiple grids have the same maximum density, one of them is randomly selected, and then one solution is randomly removed from that grid. This process is repeated until the archive size is reduced to $N_{r}$ or below.

To enhance the focus of the search process on sparse regions, a probability sampling strategy based on the reciprocal of grid density is adopted during the leader selection stage. Let O denote the set of non-empty grids. Then, the probability of selecting grid $G_{k}$ is given by:

(42) $$P(G_{k})=\frac{1/(\rho (G_{k})+\varepsilon )}{\sum_{G_{j}\in O}1/(\rho (G_{j})+\varepsilon )}$$

where ε is a small positive constant.

It can be seen that the lower the grid density, the higher the probability that the corresponding grid will be selected as the source region for leader selection. After the source grid for leader selection is determined, the corresponding solutions in that grid are selected as candidate leaders and participate in the multi-leader dynamic weighted update process described earlier. This strategy works synergistically with the previously introduced multi-leader dynamic weighted mechanism, thereby providing more representative candidate leaders for different search directions.

**Figure 3:**
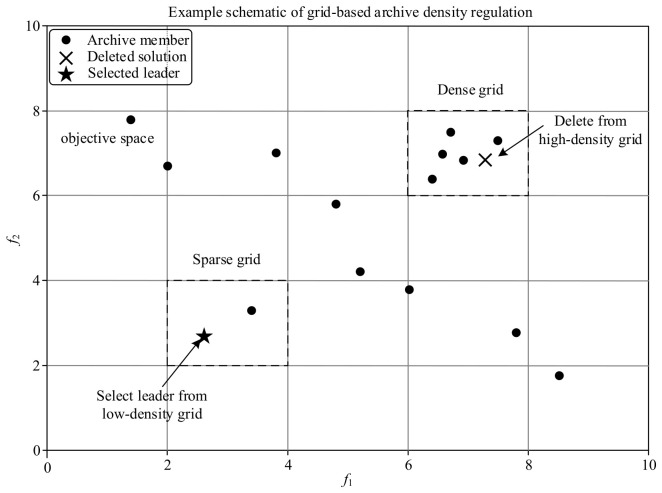
Schematic illustration of grid-based density regulation for the external archive in a two-objective space.

As illustrated in Figure 3, the grid partitioning mechanism regulates the density of the external archive through a dense-region reduction strategy and a sparse-region priority strategy. On the one hand, when the archive exceeds its capacity, solutions in high-density grids are preferentially removed to suppress local aggregation. On the other hand, during leader selection, low-density grids are given priority to enhance exploration in underexplored regions.

#### 4.3.3. Mechanism Characteristics and Performance Analysis

Compared with archive maintenance methods based on crowding distance, the proposed grid partitioning mechanism replaces local neighborhood distance estimation with discrete density statistics. This representation captures the global distribution characteristics of the solution set and enables direct identification of dense and sparse regions in the objective space. As a result, the mechanism not only mitigates local aggregation in the Pareto solution set but also provides more uniformly distributed candidate leaders for leader selection, thereby improving the overall distribution quality of solutions.

In addition, after the non-dominated solutions are identified, the grid-based method mainly involves normalization, index mapping, and density statistics. The archive maintenance process is therefore relatively simple and scalable. For 3D UAV path planning problems, this mechanism can better preserve Pareto-front coverage across multiple objectives, including path length, threat cost, smoothness cost, and altitude cost, thereby providing more stable archive support for subsequent search.

Overall, the grid-based external archive update mechanism maintains a globally balanced Pareto solution set through dense-region reduction and sparse-region priority strategies while providing more stable candidate sources for multi-leader search. Correspondingly, the three improvements proposed in this paper address three key stages of the optimization process: initial population construction, iterative search guidance, and external archive maintenance. Specifically, the AOBL initialization strategy enhances the feasibility and coverage of the initial population; the multi-leader dynamic weighting mechanism increases the diversity of search directions; and the grid-based external archive update mechanism further maintains distribution balance in the objective space. Together, these components form a collaborative optimization framework for complex 3D UAV path planning.

### 4.4. Computational Complexity Analysis

To further clarify the computational cost of the proposed IML-MOWOA, this subsection analyzes the time complexity of its main components using the notation defined in the preceding sections. Here, |M| denotes the number of obstacles in the obstacle set M, while N, $T_{\max}$, D, k, V, $N_{r}$ and $n_{g}$ retain their previously defined meanings. Since D is determined by the waypoint-based path encoding, it is proportional to the number of optimized waypoint variables.

For each candidate path, the path-length, smoothness, and altitude-cost objectives mainly require traversing the waypoint sequence, and their computational costs are O(k). The threat-cost objective requires checking the geometric relationship between each path segment and each obstacle. Since a path contains k−1 consecutive segments, the threat-cost evaluation requires O(k|M|) operations. In the four-objective UAV path planning model considered in this study, the threat-cost evaluation dominates the other path-quality objectives when obstacles are considered. Therefore, the objective evaluation cost of one candidate path can be written as O(k|M|). Accordingly, evaluating the whole population in one iteration requires O(Nk|M|).

During the initialization stage, the proposed AOBL strategy generates both the original population and the corresponding opposite population. Computing the population statistics and generating opposite samples require O(ND). Since both the original and opposite samples need to be evaluated, the objective evaluation cost in the initialization stage is O(2Nk|M|), which can be simplified to O(Nk|M|). The merged candidate set contains at most 2N individuals. The Pareto dominance comparison used to identify non-dominated candidates requires pairwise comparisons among these individuals, and each comparison involves V objectives. Therefore, the non-dominated screening cost in the initialization stage is $O(V{\left(2N\right)}^{2})=O(VN^{2})$.

If the number of adaptive adjustment cycles in the AOBL initialization stage is denoted by $I_{A}$, the initialization complexity can be expressed as $O(I_{A}(ND+Nk|M|+VN^{2}))$.

In this study, AOBL is used only during initialization, and $I_{A}$ is a fixed small number. Therefore, it does not affect the dominant iterative complexity of the algorithm. When $I_{A}$ is treated as a constant, the initialization complexity can be simplified as $O(ND+Nk|M|+VN^{2})$.

In each iteration, the multi-leader mechanism selects three representative leaders from the external archive according to grid-density information. Since the archive size is bounded by $N_{r}$, leader selection based on occupied grid statistics requires at most $O(N_{r})$ operations. In addition, computing the normalization range of the archive objective values requires $O(VN_{r})$. The weighted Chebyshev scalarization and Softmax-based dynamic weighting are performed only for three leaders over V objectives, resulting in O(V) computational cost. The fusion of the three leader decision vectors requires O(D), and the feasibility check of the composite leader, introducing one additional objective evaluation with complexity O(k|M|). Therefore, the additional computational cost introduced by the multi-leader dynamic weighting mechanism in each iteration is $O(VN_{r}+N_{r}+V+D+k|M|)$.

Compared with the population-level objective evaluation and Pareto-based archive updating, this term is lower-order and does not change the dominant iterative complexity. For the WOA-based position update, each updates a *D*-dimensional decision vector. Therefore, the position-update operation for the whole population requires O(ND). After position updating, all candidate paths are re-evaluated, which requires O(Nk|M|). Boundary handling and infeasible-solution correction are performed at the individual level. In the worst case, infeasible-solution correction may introduce additional objective evaluations for some individuals, but this cost remains linear with respect to the population size and has the same order as the population-level objective evaluation. Therefore, the dominant computational cost of the population update and evaluation stage in each iteration is O(ND+Nk|M|).

The external archive update is another major computational component. In each iteration, the current population is merged with the existing archive. Since the archive size is bounded by $N_{r}$, the number of candidate solutions to be checked is bounded by $R=N+N_{r}$. The pairwise Pareto dominance comparison over these R candidates requires $O(VR^{2})=O(V{\left(N+N_{r}\right)}^{2})$. After the non-dominated solutions are identified, the grid-based archive mechanism normalizes the objective values, assigns grid indices, and estimates grid density. These operations require $O(VR)=O(V(N+N_{r}))$.

Although the total number of possible grid cells increases as $n_{g}^{V}$, the proposed implementation does not enumerate all grid cells; it only maps the bounded archive solutions to their occupied grid cells. Therefore, the grid-based archive does not introduce an exponential time or memory term in the current implementation. Nevertheless, when the number of objectives becomes much larger, the objective space may become sparse, and the grid-density information may become less discriminative under a fixed archive size. Thus, the current validation and conclusions are limited to the four-objective formulation considered in this study, while small-scale objective extensions and many-objective cases would require further validation and, if necessary, adaptive grid resolution, objective reduction, or reference-vector-based diversity maintenance strategies.

When the temporary archive exceeds the capacity $N_{r}$, the truncation process removes solutions from the densest grid until the archive size satisfies the capacity constraint. Let E denote the number of deleted archive members in one iteration. During truncation, the temporary archive size is at most $R=N+N_{r}$, and each density-based deletion requires at most O(R) operations. Therefore, the truncation cost is O(ER). Since E≤N, this term is bounded by $O(N(N+N_{r}))$, which does not exceed the order of $O({\left(N+N_{r}\right)}^{2})$. Therefore, archive truncation does not change the dominant archive-update complexity. The dominant computational cost of the archive update stage can therefore be written as $O(V{\left(N+N_{r}\right)}^{2}+V(N+N_{r}))$, which can be simplified as $O(V{\left(N+N_{r}\right)}^{2})$. Combining the above components, the computational complexity of the main iterative process of IML-MOWOA is $O(T_{\max}[ND+Nk|M|+V{\left(N+N_{r}\right)}^{2}+VN_{r}+N_{r}+V+D+k|M|])$.

Since $VN_{r}+N_{r}+V+D+k|M|$ is lower-order compared with population-level objective evaluation and Pareto-based archive updating, the dominant iterative complexity can be expressed as $O(T_{\max}[ND+Nk|M|+V{\left(N+N_{r}\right)}^{2}])$.

Considering the AOBL-based initialization stage, the total computational complexity of IML-MOWOA is therefore $O(ND+Nk|M|+VN^{2})+O(T_{\max}[ND+Nk|M|+V{\left(N+N_{r}\right)}^{2}])$.

This analysis shows that the dominant computational cost of IML-MOWOA mainly comes from two parts: objective-function evaluation and Pareto-based archive updating. The additional operations introduced by AOBL initialization, Softmax-based multi-leader weighting, and grid-density mapping increase the computational overhead only in specific stages, but they do not change the dominant complexity order of the archive-based multi-objective optimization framework. Therefore, compared with the original MOWOA framework, IML-MOWOA improves feasible-solution generation, search guidance, and distribution maintenance while maintaining the same dominant asymptotic complexity order.

### 4.5. Overall Framework and Flowchart of the Proposed IML-MOWOA

**Figure 4:**
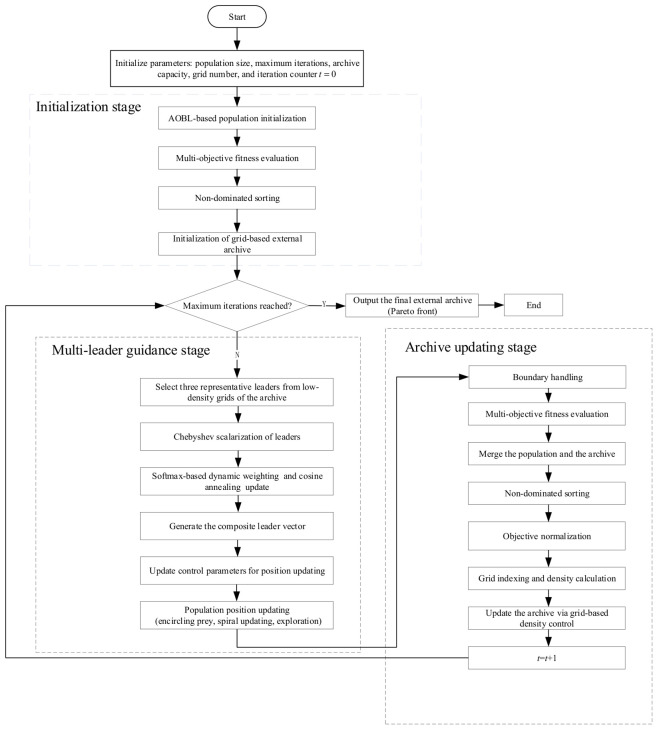
The flowchart of IML-MOWOA.

To address the multi-objective UAV path planning problem, this study proposes an improved multi-leader multi-objective whale optimization algorithm (IML-MOWOA). By integrating the adaptive opposition-based learning initialization strategy, the dynamic weighted multi-leader guidance mechanism, and the grid-based external archive update strategy, the proposed method establishes a coordinated optimization framework that improves solution feasibility, convergence stability, and distribution uniformity. The overall flowchart corresponding to the above procedure is shown in Figure 4. The main computational procedure of IML-MOWOA is summarized as follows.

Step 1: Initialization. Set the algorithmic parameters, including population size, maximum number of iterations, archive capacity, grid number, and iteration counter. Generate the initial population using the AOBL strategy. Evaluate both the original and opposite samples only during initialization, and construct the initial external archive from the resulting non-dominated solutions.

Step 2: Leader Selection and Fusion. Select three representative leaders from low-density grids in the archive, evaluate their scalarized performance using the weighted Chebyshev scalarization approach, and compute the dynamic weights through the Softmax function with adaptive cosine annealing. Then, generate the composite leader vector.

Step 3: Position Update. Update each individual using the modified WOA strategies, including encircling prey, spiral updating, and random search. After position updating, perform boundary handling and re-evaluate the updated population.

Step 4: Archive Update. Merge the updated population with the current external archive, perform non-dominated sorting, normalize the objective values, calculate grid density, and update the archive through the grid-based density control mechanism.

Step 5: Set t=t+1 and repeat Steps 2–4 until the maximum iteration number is reached.

Step 6: Output. Return the final external archive as an approximation to the Pareto-optimal solution set.

## 5. Experimental Setup and Results Analysis

To comprehensively evaluate the overall performance of the proposed IML-MOWOA in multi-objective UAV path planning, systematic comparisons are conducted with five representative multi-objective optimization algorithms, namely MOCOA [38], NSGA-II [32], MOWOA [26], MOPSO [33], and MOOOA [39], across three scenarios with different levels of environmental complexity. The comparative analysis focuses on four key aspects: convergence behavior, Pareto-front distribution uniformity, path quality at the mission-execution level, and statistical significance. This comprehensive evaluation framework is designed to assess not only the algorithms’ approximation quality in the objective space but also the feasibility and practical utility of the generated UAV paths in realistic mission scenarios.

The experimental results and corresponding analyses are presented in the following subsections. All experiments are implemented in the MATLAB R2023a environment. For fair comparison, the algorithmic parameters are uniformly configured as follows: a population size of 100, a maximum number of 500 iterations, an external archive capacity of 200, and a grid division number of 20. The sources and algorithm-specific hyperparameters of the compared methods are summarized in Table 2. In this table, $n_{\mathrm{var}}$ denotes the dimension of the decision vector, i.e., the number of optimization variables used to encode a candidate UAV path.

### 5.1. Simulation Environment and Task Scenarios

To evaluate the performance of the proposed algorithm in environments with different levels of complexity, three representative 3D UAV path planning scenarios are constructed. All simulation experiments are conducted on the basis of a digital elevation map with a resolution of 879 × 1045. Using this terrain model, three scenarios with increasing complexity—denoted as Scenario 1, Scenario 2, and Scenario 3—are constructed by configuring different numbers and spatial distributions of obstacles. The parameter settings of each scenario are summarized in Table 3, including the start and end points, obstacle locations, threat centers, and corresponding threat radii.

The coefficients of variation of obstacle height $CV_{h}$ and obstacle radius $CV_{r}$ are adopted as two quantitative indicators for evaluating environmental complexity. In addition, obstacle density is introduced as a third indicator, with a larger number of obstacles indicating a denser obstacle distribution. Meanwhile, higher values of $CV_{h}$ and $CV_{r}$ generally indicate greater variability in obstacle heights and obstacle radii, reflecting increased heterogeneity in obstacle characteristics. Such variability corresponds to more complex environments, in which UAV path planning becomes more challenging due to pronounced differences in obstacle elevation and spatial extent.

The coefficient of variation is defined as

(43) $$CV=\frac{\sigma }{\mu }$$

when σ and μ correspond to the height values of all obstacles, the height coefficient of variation $CV_{h}$ can be obtained. Similarly, by substituting the obstacle radii into Equation (43), the radius coefficient of variation $CV_{r}$ can be calculated.

As shown in Table 4, Scenario 1 exhibits the smallest values of $CV_{h}$ and $CV_{r}$, indicating the lowest environmental complexity and a relatively open environment. Scenario 2, both $CV_{h}$ and $CV_{r}$ increase compared with Scenario 1, indicating greater heterogeneity in obstacle heights and radii. Together with the increased number of obstacles, this suggests a more cluttered environment with higher complexity. Scenario 3 achieves the largest values of both coefficients and contains the highest number of obstacles, revealing the greatest environmental complexity, the densest spatial obstacle distribution, and the strongest geometric heterogeneity across all scenarios.

**Figure 5:**
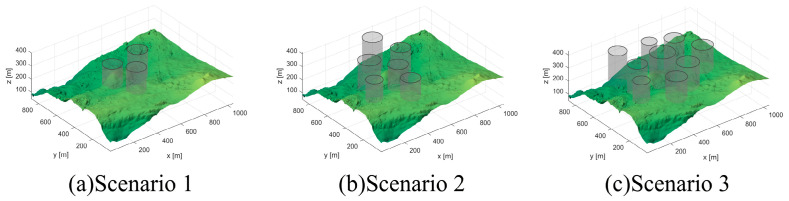
The three-dimensional drawings of three simulation environments. (**a**) Scenario 1; (**b**) Scenario 2; (**c**) Scenario 3.

The 3D terrain models corresponding to Scenario 1, Scenario 2, and Scenario 3 are illustrated in Figure 5a–c, respectively.

### 5.2. Validation Experiments on Strategy Effectiveness

#### 5.2.1. Validation of the AOBL-Based Initialization Strategy

To evaluate the efficacy of the proposed AOBL strategy in enhancing initial solution quality and feasibility, we integrate the AOBL mechanism into the MOWOA framework, resulting in a variant denoted as AMOWOA. Notably, the evaluation of opposite solutions generated by AOBL is restricted to the initialization stage. Therefore, the comparison is conducted under identical population sizes and iteration limits, while the observed performance difference mainly reflects the effect of the enhanced initialization strategy.

**Figure 6:**
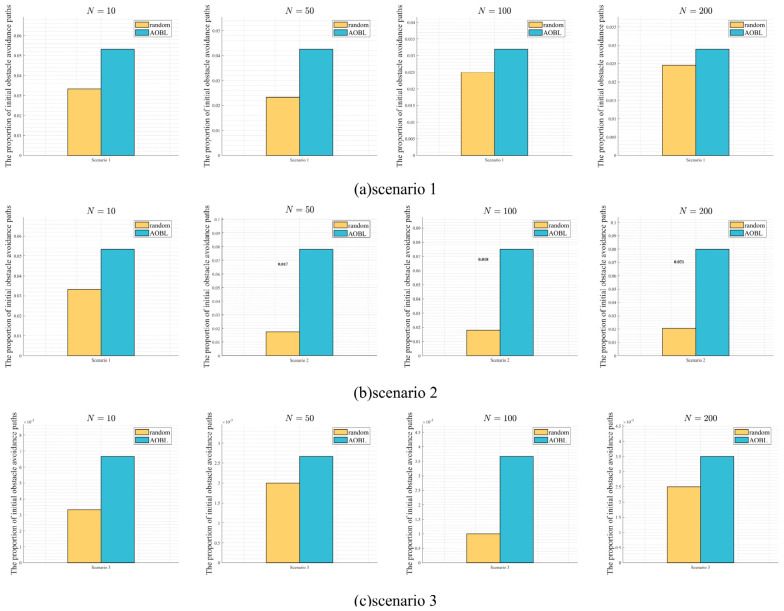
Feasible path ratios of MOWOA and AMOWOA at the initialization stage under different population sizes. (**a**) Scenario 1; (**b**) Scenario 2; (**c**) Scenario 3.

Experiments were conducted across three environments of varying complexity, with initial population sizes set to $N=\left\{10,50,100,200\right\}$. Each configuration was executed for 30 independent runs to mitigate stochastic effects. The primary performance metric is the feasible path ratio at the initialization stage, which reflects the strategy’s ability to generate valid initial solutions in complex 3D environments. The feasible path ratios obtained by MOWOA with random initialization and AMOWOA, which incorporates the AOBL-based initialization strategy, in the three scenarios are presented in Figure 6a–c.

In Scenario 1, characterized by sparse obstacles and an expansive feasible search space, AMOWOA achieves a markedly higher feasibility ratio at smaller population sizes. Although the performance gap between the two algorithms narrows as *N* increases, suggesting that larger random populations can partially improve the probability of generating feasible initial paths, AOBL still shows a clear advantage when the number of samples is limited. This result suggests that the proposed strategy reduces the dependence on large initial populations in low-complexity environments.

The advantage of AMOWOA becomes more pronounced in Scenario 2, where increased obstacle density and terrain variability raise the probability of generating infeasible paths through random initialization. Across all tested population sizes, AMOWOA consistently yields a higher feasible path ratio. This improvement can be attributed to the AOBL mechanism. By leveraging mean-guided reflection and adaptive mapping, the strategy increases the likelihood that initial samples fall into feasible regions, such as safe corridors and relatively open terrain areas. Such a mechanism improves the ability to generate feasible initial candidates and reduces the likelihood that the initial population falls into infeasible regions under moderately constrained environments.

The robustness of the AOBL strategy is most evident in Scenario 3, the most challenging urban-like environment with dense clusters of obstacles and extreme height fluctuations. In this highly constrained setting, the feasible path ratio of random initialization remains negligible because random sampling frequently produces paths that intersect obstacles or violate terrain-related constraints. In contrast, AMOWOA maintains a substantially higher feasible path ratio. By employing the AOBL-based reflection mechanism, the algorithm is more likely to generate initial paths located in narrow traversable corridors. These findings indicate that AOBL provides a clear advantage in highly constrained environments, where conventional random initialization often fails to generate feasible initial paths.

#### 5.2.2. Validation of the Multi-Leader Dynamic Weighting Mechanism

**Figure 7:**
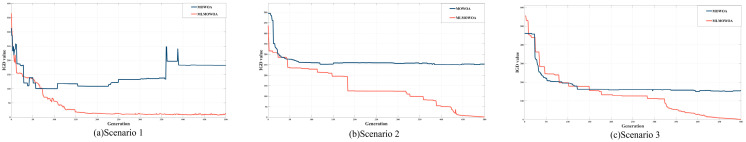
IGD convergence curves of MOWOA and MLMOWOA under the three scenarios. (**a**) Scenario 1; (**b**) Scenario 2; (**c**) Scenario 3.

To evaluate the effectiveness of the proposed multi-leader dynamic weighting mechanism in improving search diversity and convergence performance, the original MOWOA was compared with a variant termed MLMOWOA, in which the multi-leader fusion strategy was incorporated while the remaining components were kept unchanged. The IGD convergence curves of the two methods under the three scenarios are shown in Figure 7.

In the low-complexity setting of Scenario 1, MLMOWOA performs only slightly better than MOWOA. This indicates that in relatively simple environments, the original guidance strategy of MOWOA can still provide effective search direction. However, due to the limited diversity of its guidance information, MOWOA is more likely to drive the search toward restricted regions of the objective space. In contrast, MLMOWOA integrates information from multiple non-dominated solutions, thereby providing more diverse search directions and yielding more stable convergence with lower final IGD values.

The advantage of MLMOWOA becomes more pronounced in Scenario 2. MOWOA exhibits noticeable convergence stagnation during the intermediate iterations, suggesting that its search guidance becomes less effective as the problem complexity increases. By contrast, the proposed multi-leader strategy, combined with the Softmax-based weighting scheme, adaptively balances the influence of multiple guiding solutions. This helps alleviate local aggregation and maintain a better balance between convergence and diversity.

In the highly complex Scenario 3, the superiority of MLMOWOA becomes most evident. MOWOA shows a markedly slower convergence rate and a higher final IGD value, indicating weaker approximation quality under strong objective conflicts and complex constraints. By selecting leaders from different archive regions, MLMOWOA provides more balanced objective guidance. Furthermore, the Softmax-based dynamic weighting mechanism encourages more balanced guidance in the early stage and gradually assigns greater influence to higher-quality leaders in later iterations. As a result, MLMOWOA achieves better convergence stability and lower IGD values, especially in highly constrained scenarios.

#### 5.2.3. Validation of the Adaptive Cosine Annealing Mechanism

**Figure 8:**
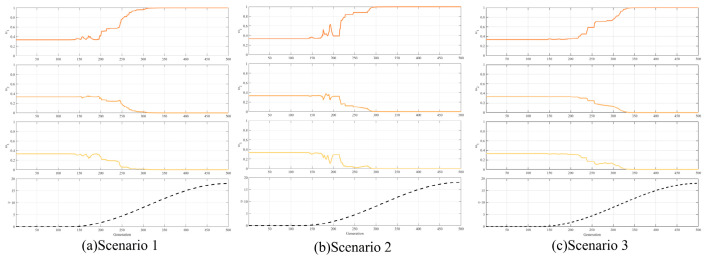
Softmax-based leader weight distributions and adaptive cosine annealing schedules under the three scenarios. (**a**) Scenario 1; (**b**) Scenario 2; (**c**) Scenario 3.

To evaluate the impact of adaptive cosine annealing on the multi-leader dynamic weighting process, we analyzed the evolution of Softmax-based leader weights together with the annealing parameter η(t) across three representative scenarios. The trajectories of the weights of three selected leaders and the evolution of η(t) are illustrated in Figure 8a–c.

As shown by the curves of η(t), this parameter remains at a low level during the early iterations, gradually increases in the intermediate stage, and reaches its maximum in the final stage. This nonlinear evolution controls the degree of discrimination in the Softmax weighting process, resulting in distinct stage-wise guidance characteristics.

During the initial optimization stage, the low value of η(t) suppresses the Softmax function’s ability to differentiate among leaders. Consequently, the leader weights in all three scenarios exhibit a near-uniform distribution, as reflected by the highly similar trajectories of the weight curves. This near-uniform weighting helps prevent the population from prematurely converging toward a single leader, thereby promoting diverse exploration of different trade-off regions in the objective space. As the search enters the middle stage, the increase in η(t) leads to a moderate differentiation among leader weights. The influence of high-quality leaders gradually increases, while that of less competitive leaders relatively decreases, yet a certain degree of diversity is still maintained. This transition facilitates a progressive shift from exploration to exploitation, helping refine the search direction while reducing the risk of premature convergence. In the final stage, as η(t) approaches its peak, the Softmax weights become increasingly concentrated on the most competitive leader. This concentration effect strengthens exploitation, steering the population toward a more consistent convergence direction and accelerating convergence toward high-quality Pareto solutions. Notably, the dominant leader differs across scenarios, indicating that the mechanism can adaptively assign guidance according to the characteristics of the problem landscape rather than relying on a fixed preference.

In summary, the adaptive cosine annealing mechanism enables a smooth transition from global exploration to focused exploitation. By dynamically regulating the influence of multiple leaders, the mechanism enhances convergence stability and improves the algorithm’s ability to approximate high-quality Pareto solutions in complex multi-objective environments.

#### 5.2.4. Sensitivity Analysis of the Number of Leaders

The number of leaders $N_{L}$ is an important parameter in the proposed multi-leader guidance mechanism. It directly affects the diversity of search directions and the amount of leader information involved in the dynamic weighted updating process. A small number of leaders may provide insufficient Pareto trade-off guidance, whereas an excessive number of leaders may introduce redundant or conflicting search information. Therefore, a sensitivity analysis of $N_{L}$ is conducted to justify the default setting used in IML-MOWOA.

In this experiment, $N_{L}$ is varied from 1 to 5, while all other parameters are kept unchanged. The IGD values obtained under the three scenarios are summarized in Table 5. For each setting, the mean and standard deviation of IGD are reported according to the same independent-run protocol used in the previous experiments.

As shown in Table 5, $N_{L}$ has a noticeable influence on the IGD performance of IML-MOWOA. When $N_{L}$ = 1, the algorithm relies on single-leader guidance, resulting in relatively high IGD values due to insufficient search-direction diversity. As $N_{L}$ increases from 1 to 3, the mean IGD decreases consistently in all three scenarios, and $N_{L}$ = 3 achieves the best results with mean IGD values of 25.4354, 26.9235, and 66.9676, respectively. However, further increasing $N_{L}$ to 4 or 5 does not improve performance and even leads to higher IGD values, especially in Scenario 3. This indicates that excessive leaders may introduce redundant or conflicting guidance. Therefore, $N_{L}$ = 3 is adopted as the default setting because it provides a suitable balance between directional diversity, convergence performance, and algorithmic stability.

#### 5.2.5. Sensitivity Analysis of the Grid Division Number

The grid division number $n_{grid}$ is an important parameter in the grid-based external archive mechanism. It determines the resolution of density estimation in the objective space and further affects archive truncation and leader selection. If $n_{grid}$ is too small, the density estimation may be too coarse to distinguish sparse and crowded Pareto regions. In contrast, an excessively large $n_{grid}$ may over-partition the objective space, making many occupied grids contain only a few solutions and weakening the discriminative ability of grid-density information. Therefore, a sensitivity analysis of $n_{grid}$ is conducted.

In this experiment, $n_{grid}$ is set to 10, 15, 20, 25, and 30, while all other parameters are kept unchanged. The results in terms of IGD and Spacing are reported in Table 6.

As shown in Table 6, the grid division number has a noticeable influence on both optimization accuracy and distribution uniformity. In Scenario 1, $n_{grid}$ = 10 obtains the lowest IGD, while $n_{grid}$ = 20 achieves the best Spacing value, indicating a more uniform distribution of non-dominated solutions. In Scenario 2, $n_{grid}$ = 20 gives the lowest IGD, and its Spacing value remains close to the best result obtained when $n_{grid}$ = 25. In Scenario 3, which is the most complex and constrained scenario, $n_{grid}$ = 20 achieves both the lowest IGD and the lowest Spacing. These results indicate that $n_{grid}$ = 20 provides the most balanced overall performance across different environmental complexities. Therefore, $n_{grid}$ = 20 is adopted as the default setting because it maintains effective density discrimination, archive diversity, and stable Pareto-front approximation quality.

#### 5.2.6. Sensitivity Analysis of θ

The parameter θ controls the duration of the initial constant-temperature phase in the adaptive cosine annealing schedule. A smaller θ causes the leader weights to become differentiated earlier, whereas a larger θ maintains nearly balanced leader weights for a longer period. Therefore, θ affects the balance between early exploration and later exploitation.

To evaluate the influence of θ, sensitivity experiments were conducted with θ = {0.1, 0.2, 0.3, 0.4, 0.5}, while all other parameters remained unchanged. The IGD results under the three scenarios are reported in Table 7.

As shown in Table 7, θ = 0.3 achieves the lowest mean IGD in all three scenarios, with values of 23.8370, 25.9358, and 62.0831, respectively. Smaller values of θ may shorten the balanced multi-leader exploration stage, while larger values may delay the transition toward more exploitative guidance. This effect is particularly evident in Scenario 3, where the mean IGD increases markedly when θ is set to 0.4 or 0.5.

#### 5.2.7. Validation of the Grid-Based External Archive Mechanism

**Figure 9:**
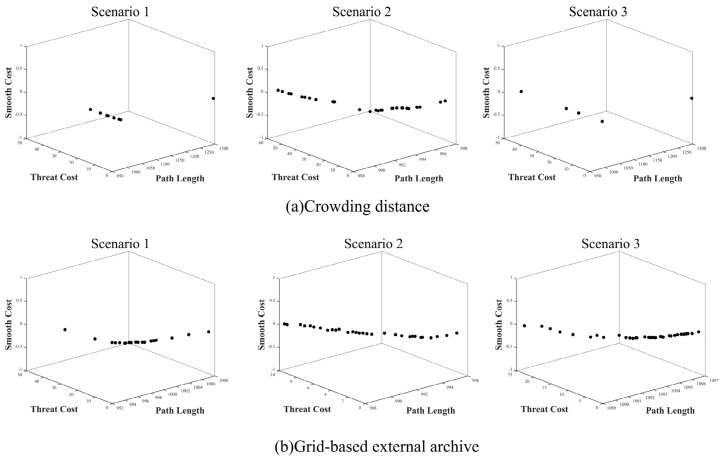
Comparison of non-dominated solution distributions obtained by the crowding-distance-based and grid-based external archive mechanisms. (**a**) Crowding distance; (**b**) Grid-based external archive.

To evaluate the efficacy of the grid-based external archive mechanism in improving the distribution uniformity and global diversity of the non-dominated solution set, we conducted a comparative experiment using the conventional crowding-distance-based archive update strategy as the baseline. The resulting non-dominated solution distributions under identical problem settings are shown in Figure 9.

As shown in Figure 9a, the non-dominated solution set obtained using the crowding-distance-based strategy exhibits noticeable clustering, with solutions densely concentrated in certain regions. Furthermore, prominent gaps appear near the boundaries and transition regions, indicating limited continuity in the approximated solution set. These observations suggest that traditional crowding-distance-based archive update mechanisms may struggle to maintain global uniformity in complex multi-objective problems, as they tend to preserve solutions in locally dense areas at the expense of overall coverage. In contrast, the non-dominated solution set generated by the grid-based mechanism (Figure 9b) exhibits better continuity and a more homogeneous distribution. The reduced presence of conspicuous clusters and sparsely populated regions suggests that grid-based density estimation can regulate solution retention more effectively. By allocating archive capacity based on grid occupancy, the strategy helps prevent excessive concentration in central regions while improving the coverage of boundary regions and extreme solutions.

These results indicate that the grid-based archive mechanism provides finer-grained control over solution density within the objective space. By discretizing the objective space into grid cells and maintaining diversity at the cell level, the proposed strategy preferentially retains solutions in underrepresented regions while reducing redundancy in overpopulated ones. Consequently, the overall uniformity and structural continuity of the approximated non-dominated solution set are improved.

In summary, the grid-based mechanism effectively alleviates the limitations of the crowding-distance-based approach. It helps maintain a well-distributed non-dominated solution set and stable diversity across different objective regions, thereby providing a more robust archive foundation for the subsequent search process of IML-MOWOA in UAV path planning.

#### 5.2.8. Ablation Study of the Proposed Components

To further evaluate the contribution of the proposed components, an ablation study was conducted based on the original MOWOA framework. Three progressive variants were considered: MOWOA1, which incorporates only the AOBL initialization strategy; MOWOA2, which further adds the multi-leader dynamic weighting mechanism; and the complete IML-MOWOA, which additionally includes the grid-based external archive update mechanism. All variants were tested under the same experimental settings, and the IGD statistics are reported in Table 8.

As shown in Table 8, the introduction of the proposed components generally improves the IGD performance of the original MOWOA. Compared with MOWOA, the complete IML-MOWOA reduces the mean IGD by 60.61%, 6.18%, and 25.04% in Scenarios 1–3, respectively. MOWOA1 achieves lower mean IGD values than MOWOA in Scenarios 1 and 2, indicating that AOBL can provide higher-quality initial candidates in low- and moderate-complexity environments. MOWOA2 further reduces the mean IGD in all three scenarios compared with MOWOA, confirming the effectiveness of the multi-leader dynamic weighting mechanism in improving search guidance. With the incorporation of the grid-based external archive update mechanism, IML-MOWOA achieves the best mean IGD in Scenarios 1 and 3 and remains highly competitive in Scenario 2. Overall, the ablation results confirm that the proposed components provide complementary improvements in convergence quality and Pareto-front approximation.

### 5.3. Comprehensive Comparative Evaluation

To rigorously assess the overall performance of IML-MOWOA in multi-objective UAV path planning, systematic benchmarking was conducted under three scenarios with increasing environmental complexity. In this evaluation, IML-MOWOA was compared with five representative multi-objective optimization algorithms. The comparison covers multiple critical dimensions: convergence behavior, the distribution uniformity of the approximated Pareto solution set, task-specific path quality, and statistical significance.

These criteria are designed to collectively quantify both the algorithms’ approximation capabilities in the objective space and their practical effectiveness in generating safe and feasible flight trajectories. Through this multi-dimensional analysis, the performance advantages of IML-MOWOA are identified and examined, thereby demonstrating its adaptability and robustness in complex environments and providing empirical support for its potential application in UAV path planning.

#### 5.3.1. Convergence and Pareto-Front Quality Analysis

**Figure 10:**
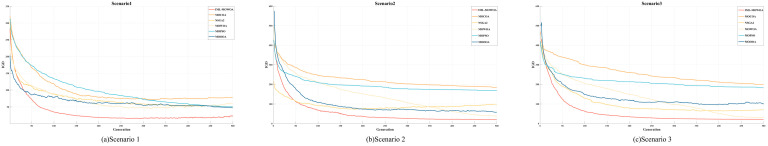
IGD convergence curves of six multi-objective algorithms under the three scenarios. (**a**) Scenario 1; (**b**) Scenario 2; (**c**) Scenario 3.

In this subsection, the convergence behavior and Pareto-front approximation quality of IML-MOWOA are evaluated by comparison with five representative multi-objective optimization algorithms. IGD is first used to evaluate the convergence accuracy of the obtained non-dominated solution sets. To avoid relying on a single quality indicator, HV and Spacing are further introduced as complementary metrics. HV evaluates the comprehensive quality of convergence and objective-space coverage, where a larger value indicates better Pareto-front approximation. Spacing measures the distribution uniformity of non-dominated solutions, where a smaller value indicates a more even distribution along the approximated Pareto front. Figure 10 illustrates the IGD convergence trajectories under the three scenarios, while Table 9 summarizes the IGD statistics obtained from 30 independent runs. Table 10 further reports the HV and Spacing results of the six algorithms.

Across all scenarios, IML-MOWOA consistently demonstrates superior convergence performance. Its IGD curves exhibit a steeper descent during the initial phase and settle at significantly lower final values, reflecting advantages in both convergence speed and approximation quality. Furthermore, the standard deviations of IML-MOWOA remain at a competitive level in all scenarios, indicating good stability and robustness relative to the competing metaheuristics.

The 95% confidence interval was calculated as

(44) $$\bar{x}\pm t_{\left(0.975,n-1\right)}\frac{s}{\sqrt{n}}$$

where $\bar{x}$, s, and n denote the mean, standard deviation, and number of independent runs, respectively.

In Scenario 1, all algorithms exhibit a decreasing IGD trend as the number of iterations increases, indicating that the approximated Pareto solution sets gradually move toward the reference front. However, IML-MOWOA shows a faster decline in the early search stage and maintains the lowest IGD value during the later iterations. As reported in Table 9, IML-MOWOA obtains the lowest mean IGD of 23.8370 with a relatively small standard deviation, demonstrating better convergence accuracy and stability than the competing algorithms. This advantage is also supported by the HV and Spacing results. IML-MOWOA achieves the highest HV value of 1.4091 and the lowest Spacing value of 0.0352, indicating that the obtained non-dominated solutions have better objective-space coverage and a more uniform distribution. Although the HV improvement over NSGA-II is relatively small in this low-complexity scenario, the combined results of IGD, HV, and Spacing show that IML-MOWOA achieves a more balanced Pareto-front approximation in terms of convergence accuracy, coverage, and distribution uniformity.

The performance divergence becomes more pronounced in Scenario 2 as the environmental complexity increases. The IGD values of MOCOA and MOPSO increase to 174.14 and 178.49, respectively, indicating severe degradation in convergence quality under denser obstacles and more constrained search conditions. Although NSGA-II and MOWOA remain more competitive in this scenario, neither achieves better overall Pareto-front quality than IML-MOWOA. Specifically, IML-MOWOA obtains the lowest mean IGD of 25.94, indicating the best average convergence accuracy among the six algorithms. It should be noted that the IGD improvement over MOWOA is relatively moderate; however, the additional HV and Spacing results further demonstrate the advantage of IML-MOWOA. IML-MOWOA achieves the highest HV value of 1.4057 and the lowest Spacing value of 0.0436, suggesting better objective-space coverage and a more uniform distribution of non-dominated solutions. Therefore, in Scenario 2, the advantage of IML-MOWOA is reflected not only in convergence accuracy but also in its ability to maintain balanced Pareto-front coverage and distribution quality under increased environmental complexity.

In the most challenging Scenario 3, where extreme obstacle density and height variability impose strict constraints, the performance of traditional algorithms deteriorates sharply. MOPSO and MOCOA yield average IGD values of 239.57 and 218.14, respectively, indicating poor approximation quality in this highly constrained scenario. Although NSGA-II, MOWOA, and MOOOA achieve markedly better results than MOCOA and MOPSO, their terminal IGD values remain considerably higher than that of IML-MOWOA. IML-MOWOA obtains the lowest mean IGD of 62.08, indicating superior convergence accuracy under severe objective conflicts and narrow feasible regions. This advantage is further supported by the HV and Spacing results. IML-MOWOA achieves the highest HV value of 1.3689 and the lowest Spacing value of 0.0126, demonstrating better objective-space coverage and a more uniform distribution of non-dominated solutions. These results suggest that the multi-leader guidance mechanism and the grid-based external archive jointly improve the robustness of Pareto-front approximation in complex environments by guiding the search toward diverse trade-off regions while regulating solution distribution.

In summary, IML-MOWOA demonstrates the most stable overall Pareto-front approximation performance among the compared algorithms. The lower IGD values indicate better convergence accuracy, the higher HV values reflect stronger objective-space coverage, and the lower Spacing values demonstrate a more uniform distribution of non-dominated solutions. These advantages become more evident as the environmental complexity increases, suggesting that the proposed multi-leader guidance and grid-based archive mechanisms are effective in maintaining convergence quality, coverage, and distribution uniformity in constrained 3D UAV path planning scenarios.

#### 5.3.2. Distribution Analysis of Pareto Solution Sets

**Figure 11:**
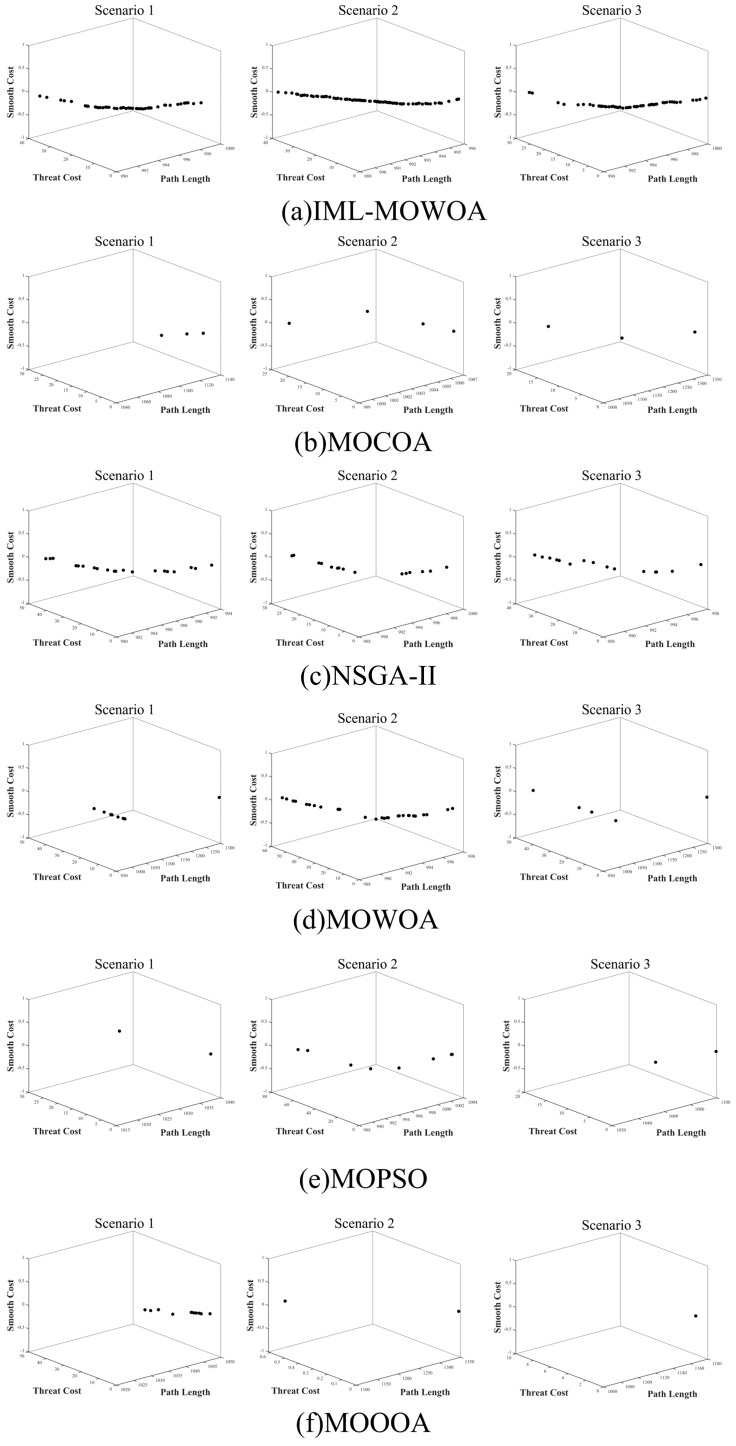
Non-dominated solution distributions of six multi-objective optimization algorithms under three different scenarios. (**a**) IML-MOWOA; (**b**) MOCOA; (**c**) NSGA-II; (**d**) MOWOA; (**e**) MOPSO; (**f**) MOOOA.

To further evaluate the algorithms’ ability to maintain distribution uniformity, coverage, and structural continuity, a visual comparative analysis of the resulting non-dominated solution sets was conducted. Figure 11 illustrates the non-dominated solution distributions generated by IML-MOWOA and the five baseline algorithms under the three experimental scenarios.

The results indicate that as obstacle density and height variability increase, the distribution of the approximated non-dominated solutions becomes progressively more irregular and fragmented, posing increasing challenges to solution uniformity. Under these increasingly restrictive conditions, the differences in distribution quality among the algorithms become increasingly apparent.

In the low-complexity Scenario 1, although most algorithms cover the main regions of the approximated solution set, clear differences in distribution uniformity can still be observed. IML-MOWOA yields a more balanced and continuous solution distribution, indicating that the combination of multi-leader guidance and grid-based archive control is beneficial for maintaining distribution uniformity. By contrast, the solution sets obtained by MOCOA, MOWOA, and MOOOA tend to cluster in central regions, suggesting limitations in crowding-distance-based density estimation when maintaining uniform solution distributions. MOPSO shows a fragmented distribution with conspicuous gaps, indicating limited coverage in certain regions, while NSGA-II, although acceptable overall, still exhibits moderate clustering near boundary regions compared with IML-MOWOA.

The distributional performance gap widens further in Scenario 2 as environmental complexity increases. IML-MOWOA maintains relatively comprehensive coverage across the approximated solution set and preserves better structural consistency even in sparse and transitional regions. This results in a more balanced and representative distribution of trade-off solutions. In contrast, MOCOA and MOPSO exhibit severe fragmentation, with multiple missing regions indicating insufficient coverage under heightened objective conflicts. Although NSGA-II and MOWOA preserve the basic structure of the approximated solution set, their distributions still exhibit evident clustering. These observations suggest that IML-MOWOA is more effective at balancing the search distribution across the objective space, thereby improving both continuity and uniformity.

In the most challenging Scenario 3, characterized by extreme obstacle density and highly complex objective interactions, the degradation of conventional algorithms becomes most evident. IML-MOWOA consistently preserves the primary structure of the approximated solution set, maintaining relatively stable solution density in both dense and sparse regions and forming a more complete and homogeneous set. By contrast, MOCOA, MOPSO, and MOOOA exhibit severe distribution deterioration, with substantial regions of the approximated solution set left uncovered. While NSGA-II and MOWOA achieve partial coverage, they still exhibit obvious density biases and noticeable discontinuities near the boundary and transition regions.

Overall, the distribution maintenance performance of the traditional algorithms deteriorates markedly as scenario complexity increases. In contrast, IML-MOWOA consistently maintains better continuity and coverage of the approximated solution set across all test cases. These findings support the effectiveness of the grid-based external archive strategy in alleviating local concentration and promoting a more globally balanced distribution in complex multi-objective landscapes.

#### 5.3.3. Task-Level Performance Evaluation

**Figure 12:**
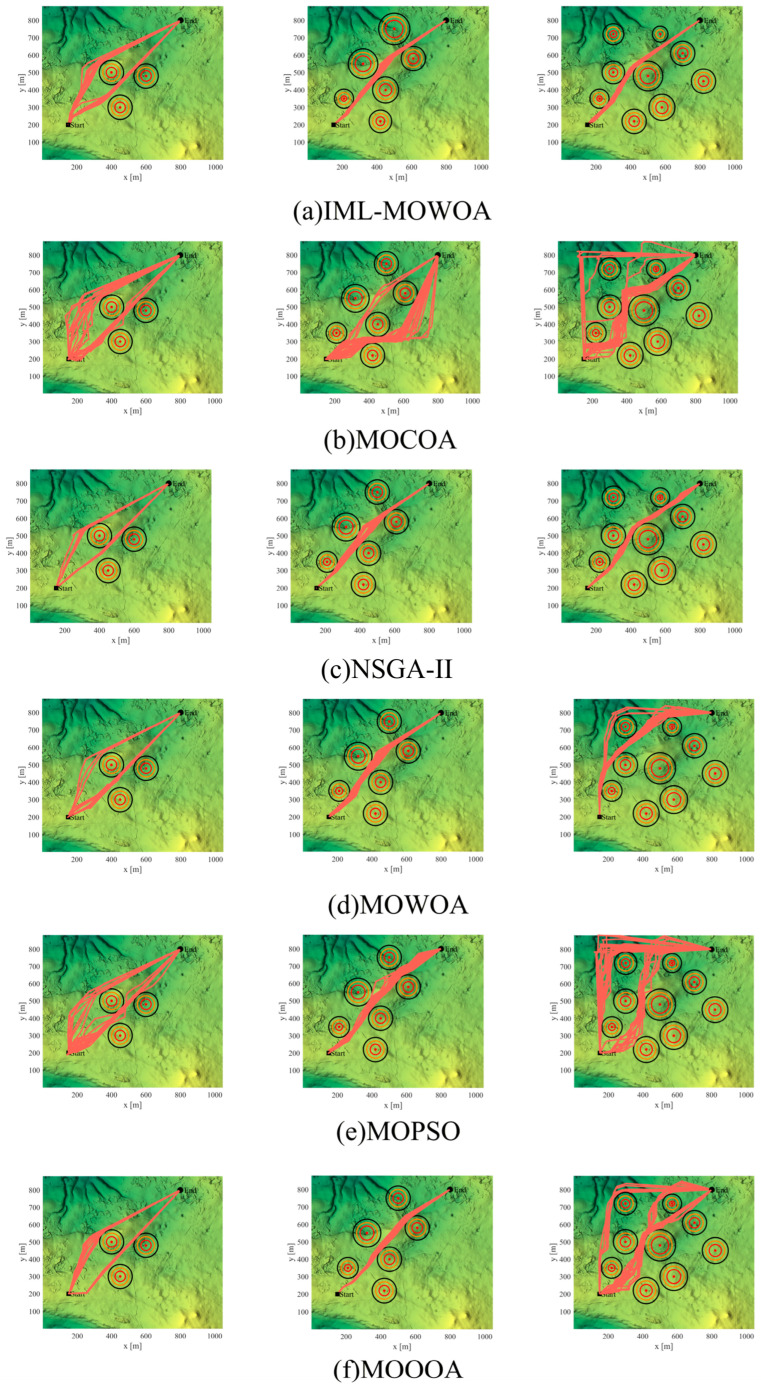
Top-view trajectories obtained by six algorithms under the four-objective formulation across three scenarios. (**a**) IML-MOWOA; (**b**) MOCOA; (**c**) NSGA-II; (**d**) MOWOA; (**e**) MOPSO; (**f**) MOOOA.

**Figure 13:**
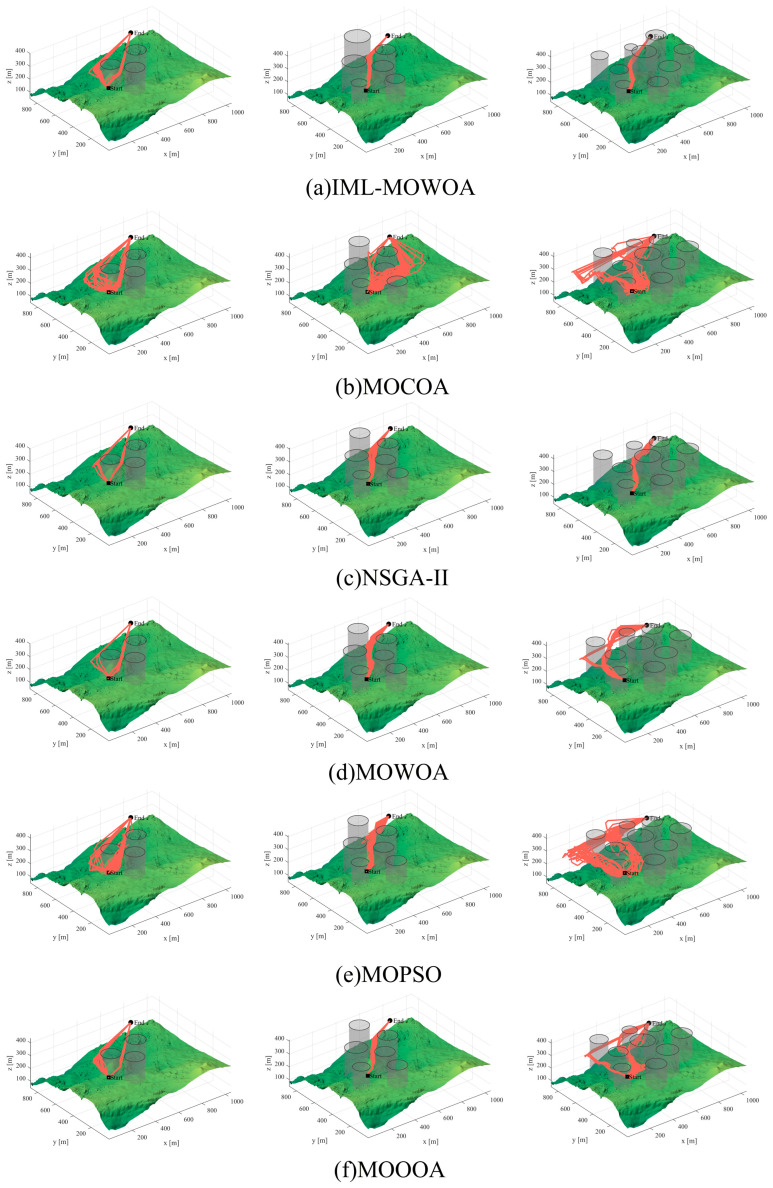
Three-dimensional views of trajectories obtained by six algorithms under the four-objective formulation across three scenarios. (**a**) IML-MOWOA; (**b**) MOCOA; (**c**) NSGA-II; (**d**) MOWOA; (**e**) MOPSO; (**f**) MOOOA.

This subsection evaluates the algorithms’ performance using practical UAV path planning metrics, including path length $f_{1}$, threat cost $f_{2}$, smoothness cost $f_{3}$, and altitude cost $f_{4}$. A systematic comparison was conducted across three scenarios of varying complexity. The geometric characteristics, obstacle avoidance behavior, and flight safety of the generated trajectories are visualized in both top-view and 3D perspectives, as shown in Figure 12 and Figure 13, with quantitative statistics summarized in Table 11.

In Scenario 1, which is characterized by relatively low environmental complexity, all algorithms are able to generate feasible paths; however, clear performance differences still emerge. IML-MOWOA produces the largest number of feasible solutions, exceeding the best-performing competing algorithm, MOOOA, by 22.03% in terms of feasible-path count, reflecting its strong ability to maintain feasibility even in less constrained environments. The resulting trajectories are characterized by smooth curvature and proactive obstacle avoidance, leading to the lowest smoothness cost and altitude cost among all compared algorithms. Specifically, the mean smoothness cost of IML-MOWOA is 64.71% lower than that of the next-best competing algorithm, NSGA-II, while its mean altitude cost is 5.03% lower than that of MOOOA. It should be noted that, in this relatively simple scenario, NSGA-II obtains a slightly shorter average path length, while MOPSO reports a lower average threat cost. Nevertheless, these isolated metric-level advantages do not translate into a more balanced overall path quality. By contrast, IML-MOWOA maintains a larger feasible-solution set while simultaneously achieving superior smoothness and altitude stability. Therefore, its advantage in Scenario 1 is better interpreted as a balanced multi-objective trade-off rather than absolute dominance in every single metric.

The performance gap widens in Scenario 2 as obstacle density and terrain variability increase. IML-MOWOA maintains the highest number of feasible paths in Scenario 2, exceeding the best-performing competing algorithm, MOOOA, by 30.06% in feasible-path count. It also achieves the lowest mean values for all four task-level cost metrics. Compared with the best competing algorithm for each corresponding metric, IML-MOWOA reduces the mean path length by 1.03%, threat cost by 41.00%, smoothness cost by 63.24%, and altitude cost by 27.67%. This indicates that the proposed improvement mechanisms are beneficial under moderate constraints. Its trajectories reflect a sophisticated trade-off between efficiency, safety, and smoothness, while maintaining stable altitude profiles. In contrast, MOCOA and MOPSO show less favorable altitude-control performance, as reflected by their substantially higher altitude costs. MOPSO also exhibits a relatively large smoothness cost, suggesting that it has difficulty maintaining stable trajectory quality under moderate environmental constraints. While NSGA-II and MOOOA achieve moderate results, their overall task-level performance remains less balanced than that of IML-MOWOA, particularly in terms of threat avoidance, smoothness, and altitude stability. Overall, the results in Scenario 2 demonstrate that IML-MOWOA provides not only a larger feasible-solution set but also a more consistent multi-objective balance across the four path-quality indicators.

In the most challenging Scenario 3, marked by dense obstacle clusters and extreme height fluctuations, IML-MOWOA demonstrates a more pronounced overall advantage. It identifies the largest number of feasible paths in Scenario 3, exceeding the best-performing competing algorithm, NSGA-II, by 234.97%, underscoring its strong adaptability to highly constrained environments. Moreover, IML-MOWOA achieves the lowest average path length, threat cost, smoothness cost, and altitude cost in this scenario. Compared with the best competing algorithm for each corresponding metric, IML-MOWOA reduces the mean path length by 1.65%, threat cost by 28.45%, smoothness cost by 53.23%, and altitude cost by 29.88%. These results indicate that its advantage becomes more evident as the feasible search space becomes increasingly sparse. As evidenced by the 3D trajectory visualizations, IML-MOWOA effectively navigates narrow yet traversable corridors while ensuring fluid transitions and reliable threat mitigation. Conversely, competing algorithms struggle to sustain stable path quality; many yield trajectories with abrupt vertical maneuvers or inefficient detours that substantially inflate cost values. In this context, the grid-based archive mechanism is likely to play an important role by preserving a more diverse pool of feasible candidates for sustained exploration in the complex landscape.

Overall, the results show that IML-MOWOA provides a more reliable and balanced trade-off among path feasibility, flight safety, trajectory smoothness, and altitude stability, especially as environmental complexity increases. Although a few isolated metrics in the low-complexity scenario are achieved by baseline algorithms, IML-MOWOA exhibits stronger overall robustness and consistency across scenarios. These results support its practical effectiveness in balancing conflicting objectives under representative UAV path planning scenarios and demonstrate its advantages in handling complex environmental constraints.

#### 5.3.4. Statistical Significance Analysis

To rigorously determine whether the performance advantages of IML-MOWOA are statistically significant, we performed the Wilcoxon rank-sum test on the quality metrics computed from the feasible paths obtained in all three scenarios. The significance level was set at α = 0.05, with IML-MOWOA used as the reference algorithm. The pairwise comparisons against MOCOA, NSGA-II, MOWOA, MOPSO, and MOOOA are summarized in Table 12. In the statistical reporting, the symbols “+”, “−”, and “=” indicate that IML-MOWOA is significantly better than, significantly worse than, or not significantly different from the competing algorithm, respectively.

The statistical test includes path length, threat cost, smoothness cost, altitude cost, and IGD. As presented in Table 12, IML-MOWOA achieves the “+” result in most pairwise comparisons across the three scenarios, and no statistically significantly worse result is observed. This indicates that the performance advantages of IML-MOWOA are reflected across multiple evaluation dimensions, including task-level path quality and Pareto-front approximation quality.

Overall, the Wilcoxon rank-sum test results demonstrate that IML-MOWOA achieves statistically reliable advantages across most evaluation metrics and comparison algorithms. No statistically significant worse result is observed, indicating that the proposed method maintains stable competitiveness while improving task-level path quality and Pareto-front approximation. Although a small number of comparisons show no significant difference, these cases mainly occur for isolated metrics and do not weaken the overall statistical evidence. The results therefore support the conclusion that IML-MOWOA provides a more robust and balanced multi-objective optimization performance under different 3D UAV path planning scenarios.

### 5.4. Discussion

The experimental results across diverse scenarios collectively demonstrate that IML-MOWOA consistently surpasses the benchmark algorithms in convergence behavior, the distribution of the approximated Pareto solution set, and task-level performance. These advancements are not the result of isolated modifications; rather, they emerge from the synergistic interaction between the AOBL initialization, the multi-leader dynamic weighting mechanism, the adaptive cosine annealing scheme, and the grid-based external archive strategy. This integrated framework significantly enhances the algorithm’s adaptability to the complexities inherent in UAV path planning.

First, the convergence analysis indicates that IML-MOWOA achieves faster IGD reduction and better approximation quality of the Pareto solution set. This suggests that the multi-leader fusion mechanism effectively mitigates the inherent limitations of the standard WOA, which is often hampered by unidirectional search trajectories and local stagnation in multi-objective landscapes. Concurrently, the adaptive cosine annealing mechanism dynamically adjusts the Softmax scaling parameter, thereby promoting broader exploration in the early stage before progressively shifting toward more focused exploitation. This transition helps improve convergence stability while preserving population diversity.

Second, the distribution analysis reinforces the advantage of IML-MOWOA in preserving solution diversity. By substituting local crowding-distance metrics with global grid-based density statistics, the archive mechanism adaptively retains solutions in sparse regions while curbing excessive clustering. Consequently, IML-MOWOA maintains better structural continuity and broader coverage of the approximated non-dominated solution set, thereby reducing the local collapse and uneven distribution often observed in conventional methods.

Furthermore, task-level comparisons reveal that IML-MOWOA achieves better trade-offs among key UAV objectives while yielding a significantly larger number of feasible trajectories. The AOBL-based initialization provides a higher-quality initial solution pool, allowing the search to reach feasible regions more efficiently even in complex terrains. Moreover, the synergy between the multi-leader structure and the grid-based archive helps maintain smoother and more coherent paths, particularly in narrow corridors and regions with steep terrain variations, where IML-MOWOA consistently produces safer and more terrain-adaptive trajectories.

Finally, the Wilcoxon rank-sum test provides statistical support for the observed performance differences in IML-MOWOA under the tested scenarios. These results indicate that the reported performance gains are statistically robust and unlikely to be artifacts of stochastic variation. This empirical evidence further substantiates the algorithm’s efficacy in handling the intricate trade-offs required for multi-objective UAV navigation.

In summary, the convergence stability, distribution uniformity, and task-level advantages of IML-MOWOA are rooted in the coordinated operation of its core components. The findings suggest that IML-MOWOA provides a competitive optimizer-level framework for the considered offline static 3D UAV path planning problem, while its applicability to dynamic and physically realistic UAV missions requires further investigation.

## 6. Conclusions

This study addresses the critical challenges of solution feasibility, convergence efficiency, and distribution uniformity of the non-dominated solution set in multi-objective UAV path planning within complex 3D environments. We propose an enhanced multi-objective whale optimization algorithm, IML-MOWOA, which systematically refines the conventional MOWOA framework in three aspects: population initialization, multi-directional search guidance, and archive maintenance. Specifically, the AOBL strategy was introduced to improve the quality of initial samples and the coverage of feasible regions. A multi-leader dynamic weighting mechanism, integrated with an adaptive cosine annealing scheme, was developed to enhance directional diversity and stage-adaptive search capability. Finally, a grid-based external archive update strategy was incorporated to provide finer global control over the distribution of the approximated non-dominated solution set.

Extensive benchmarking under 3D scenarios with varying levels of complexity indicates that IML-MOWOA achieves competitive overall performance compared with five representative multi-objective optimization algorithms. Within the considered offline static planning setting, IML-MOWOA shows improved convergence behavior, better Pareto-front distribution, and a favorable balance among task-level path-quality metrics. In highly constrained environments with dense obstacles and severe terrain variability, the proposed method is able to generate comparatively shorter, smoother, and safer trajectories while maintaining a high number of feasible paths. The Wilcoxon rank-sum tests further support the statistical significance of the observed performance differences for the reported metrics.

The proposed method is limited to static offline planning and does not directly handle moving obstacles, newly emerging threats, or online perception uncertainty. In partially dynamic or uncertain environments, the impact on planning performance would depend on the magnitude and location of environmental changes. Minor changes far from the planned trajectory may have limited influence on path feasibility but may affect the optimality margin, whereas newly appearing obstacles or threats near the planned corridor may increase the threat cost, reduce feasibility, or even violate safety constraints. Therefore, the present results should not be directly generalized to dynamic mission scenarios without an additional online replanning module.

In summary, IML-MOWOA provides a robust and effective optimization framework for handling strongly conflicting objectives in complex 3D environments. Future research will focus on integrating IML-MOWOA with deep learning-based environment modeling, dynamic threat prediction, and distributed multi-UAV cooperative frameworks. Such advancements aim to enhance the algorithm’s adaptability and real-time applicability in dynamic mission planning and multi-agent coordination scenarios.

## Figures and Tables

**Table 1:** Comparison of representative path planning and multi-objective optimization methods.

Method/Study	Category	Main Advantages	Main Limitations
A* and simulated annealing [8,9]	Classical static planning	Simple structure; clear search logic	Limited scalability in complex 3D multi-objective planning
PSO, ACO, GWO, WHO, and COA-based methods [14,15,16,17,18]	Swarm intelligence planning	Flexible nonlinear search; strong heuristic capability	Rely on weighted single-objective formulations
NSGA-II and MOPSO [32,33]	Multi-objective evolutionary	Pareto-solution generation without predefined weights	Possible convergence instability and uneven solution distribution
MOWOA [26]	Multi-objective whale optimization	Simple structure; archive-based Pareto optimization	Low feasible-solution ratio; single-leader search bias
RMOWOA, LE-MOWOA, and MOWOA/D [28,29,30]	Improved MOWOA variants	Improved convergence, diversity, or decomposition-based search	Not tailored to constrained static 3D UAV path planning
IML-MOWOA	Proposed method	Feasibility-enhanced initialization; multi-leader guidance; grid-based archive maintenance	Dynamic replanning and moving obstacles are not considered

**Table 2:** Sources and algorithm-specific hyperparameter settings of the compared algorithms.

Algorithm	Source	Algorithm-Specific Hyperparameters	Values
IML-MOWOA		Number of leaders $N_{L}$; spiral shape constant b; Softmax temperature bounds $\eta_{\min}$, $\eta_{\max}$; constant-temperature phase ratio θ	$N_{L}$ = 3; $\eta_{\min}$ = $10^{-3}$; $\eta_{\max}$ = 12; θ = 0.3
MOCOA	[38]	Resource-selection probability $p_{r}$; maximum exploration scaling factor $\lambda_{\max}$; cleaning-factor lower bound $\beta_{\min}$; cleaning-factor amplitude $\beta_{\Delta }$; stochastic personal-best update probability $p_{b}$	$p_{r}$ = 0.5; $\lambda_{\max}$ = 0.5; $\beta_{\min}$ = 0.5; $\beta_{\Delta }$ = 0.5; $p_{b}$ = 0.5
NSGA-II	[32]	Crossover probability $p_{c}$; mutation probability $p_{m}$; crossover distribution index $\eta_{c}$; mutation distribution index $\eta_{m}$	$p_{c}$ = 0.9; $p_{m}$ = 1/$n_{\mathrm{var}}$; $\eta_{c}$ = $\eta_{m}$ = 20
MOWOA	[26]	Convergence factor a; spiral shape constant b; position-update switching probability p; stochastic personal-best update probability $p_{b}$	$a=2-\frac{2t}{T_{\max}}$; b = 1; p = 0.5; $p_{b}$ = 0.5
MOPSO	[33]	Inertia weight W; personal confidence factor $C_{1}$; swarm confidence factor $C_{2}$; uniform mutation percentage $u_{mut}$; non-uniform mutation decay exponent γ;	W = 0.4; $C_{1}$ = $C_{2}$ = 2; $u_{mut}$ = 0.5; γ = 5$n_{\mathrm{var}}$
MOOOA	[39]	Circle chaotic map parameters $a_{c},b_{c},c_{c}$; fish-selection probability $p_{f}$; boundary-repair switching probability $p_{r}$; stochastic personal-best update probability $p_{b}$	$a_{c}$ = 0.556; $b_{c}$ = 0.16; $c_{c}$ = 0.2; $p_{f}$ = 0.5; $p_{r}$ = 0.5; $p_{b}$ = 0.5

**Table 3:** The parameters for three environments.

Scenario	Start Point	End Point	Obstacles	Obstacle Center	Obstacle Radius
Scenario 1	(150, 200, 150)	(900, 800, 150)	3	(400, 500, 100) (600, 480, 150) (450, 300, 150)	70 70 70
Scenario 2	(150, 200, 150)	(900, 800, 150)	6	(210, 350, 110) (420, 220, 118) (610, 580, 126) (450, 400, 138) (320, 550, 152) (500, 750, 185)	55 65 70 75 85 90
Scenario 3	(150, 200, 150)	(900, 800, 150)	9	(220, 350, 100) (420, 220, 110) (580, 300, 140) (300, 500, 120) (500, 480, 200) (700, 610, 200) (300, 720, 130) (570, 720, 100) (820, 450, 120)	55 70 75 65 85 70 60 45 70

**Table 4:** Coefficients of variation $CV_{h}$ and $CV_{r}$ for three simulation environments.

Scenario	$CV_{h}$	$CV_{r}$
Scenario 1	0.176	0.000
Scenario 2	0.180	0.160
Scenario 3	0.270	0.166

**Table 5:** Sensitivity analysis of the number of leaders $N_{L}$ in terms of IGD under the three scenarios.

Scenario		$N_{L}$ = 1	$N_{L}$ = 2	$N_{L}$ = 3	${Ν}_{L}$ = 4	$N_{L}$ = 5
Scenario 1	Mean	39.3935	29.4356	25.4354	31.8254	43.2079
	Std	21.9281	12.6228	6.8187	11.5183	26.8187
Scenario 2	Mean	62.0069	34.2662	26.9235	40.0317	64.6246
	Std	34.9609	19.0691	11.9246	18.3453	20.1284
Scenario 3	Mean	89.7592	76.7512	66.9676	71.1066	121.5070
	Std	38.2642	31.0614	21.7369	29.3241	30.5178

**Table 6:** Sensitivity analysis of the grid division number $n_{grid}$ in terms of IGD and Spacing under the three scenarios.

Scenario		$n_{grid}$ = 10	$n_{grid}$ = 15	$n_{grid}$ = 20	$n_{grid}$ = 25	$n_{grid}$ = 30
Scenario 1	IGD	19.0863	29.7609	24.2919	27.0236	29.7748
	Spacing	0.0476	0.0401	0.0352	0.0398	0.0416
Scenario 2	IGD	33.7017	31.3425	27.9367	31.0072	41.4425
	Spacing	0.1024	0.0843	0.0414	0.0388	0.0510
Scenario 3	IGD	80.8615	72.7530	61.7533	68.9742	98.8287
	Spacing	0.1327	0.0851	0.0273	0.0513	0.1140

**Table 7:** Sensitivity analysis of θ in terms of IGD under the three scenarios.

Scenario		θ = 0.1	θ = 0.2	θ = 0.3	θ = 0.4	θ = 0.5
Scenario 1	Mean	30.7780	26.7742	23.8370	24.7742	26.6153
	Std	17.9713	15.2474	7.8398	8.0075	12.1808
Scenario 2	Mean	37.8095	29.1031	25.9358	37.5741	47.1058
	Std	20.0002	21.2044	14.7139	14.3051	12.8310
Scenario 3	Mean	104.8864	76.4727	62.0831	128.7535	230.2248
	Std	34.5095	27.5687	13.4621	21.3420	30.9396

**Table 8:** Ablation results of the proposed components in terms of IGD under the three scenarios.

Scenario		MOWOA	MOWOA1	MOWOA2	IML-MOWOA
Scenario 1	Mean	60.5091	44.5807	35.1749	23.8370
	Std	46.8848	17.5028	11.3572	7.8398
	95% CI	[43.0020, 78.0162]	[38.0450, 51.1164]	[30.9341, 39.4157]	[20.9096, 26.7644]
Scenario 2	Mean	27.6442	27.1728	25.6919	25.9358
	Std	3.5907	9.8622	16.8848	14.7139
	95% CI	[26.3034, 28.9850]	[23.4902, 30.8554]	[19.3870, 31.9968]	[20.4415, 31.4301]
Scenario 3	Mean	82.8266	86.3119	68.2172	62.0831
	Std	31.0506	30.3583	24.9436	13.4621
	95% CI	[71.2321, 94.4211]	[74.9759, 97.6479]	[58.9031, 77.5313]	[57.0563, 67.1099]

**Table 9:** IGD statistics, including mean, standard deviation, and 95% confidence interval, of the non-dominated solution sets obtained by six multi-objective optimization algorithms under the three scenarios.

Scenario		IML-MOWOA	MOCOA	NSGA-II	MOWOA	MOPSO	MOOOA
Scenario 1	Mean	23.8370	77.3993	51.0095	60.5091	50.4636	47.2004
	Std	7.8398	21.4333	22.3417	46.8848	11.6399	21.0125
	95% CI	[20.9096, 26.7644]	[69.3960, 85.4026]	[42.6670, 59.3520]	[43.0020, 78.0162]	[46.1172, 54.8100]	[39.3542, 55.0466]
Scenario 2	Mean	25.9358	174.1352	89.6281	27.6442	178.4892	51.4464
	Std	14.7139	17.7530	24.2465	3.5907	16.5655	10.0640
	95% CI	[20.4415, 31.4301]	[167.5061, 180.7643]	[80.5743, 98.6819]	[26.3034, 28.9850]	[172.3035, 184.6749]	[47.6884, 55.2044]
Scenario 3	Mean	62.0831	218.1417	85.6589	82.8266	239.5655	89.8490
	Std	13.4621	47.6887	34.6454	31.0506	26.3569	26.7100
	95% CI	[57.0563, 67.1099]	[200.3344, 235.9490]	[72.7221, 98.5957]	[71.2321, 94.4211]	[229.7237, 249.4073]	[79.8753, 99.8227]

**Table 10:** HV and Spacing results of the non-dominated solution sets obtained by six multi-objective optimization algorithms under the three scenarios.

Scenario		IML-MOWOA	MOCOA	NSGA-II	MOWOA	MOPSO	MOOOA
Scenario 1	HV	1.4091	1.2459	1.4026	1.3063	1.2653	1.3907
	Spacing	0.0352	0.0434	0.0583	0.0423	0.0396	0.0375
Scenario 2	HV	1.4057	1.2474	1.3546	1.2840	1.1691	1.2926
	Spacing	0.0436	0.0621	0.0628	0.0988	0.1019	0.1680
Scenario 3	HV	1.3689	0.9908	1.2123	1.2706	1.0029	1.2346
	Spacing	0.0126	0.0778	0.0267	0.0264	0.0817	0.0366

**Table 11:** Number of feasible paths and statistics (mean and standard deviation) of path costs obtained by six multi-objective optimization algorithms under the three scenarios.

Scenario		Algorithm						
			IML-MOWOA	MOCOA	NSGA-II	MOWOA	MOPSO	MOOOA
Scenario 1	Total number		5943	1321	4694	4157	1653	4870
	Path length	Mean	989.57	1068.64	986.56	1002.60	1057.54	1011.65
		Std	7.95	70.38	26.54	23.59	242.07	116.63
	Threat cost	Mean	2.45	2.55	5.84	6.49	1.45	8.14
		Std	2.16	5.19	6.34	5.79	2.27	6.53
	Smooth cost	Mean	0.66	9.06	1.87	10.30	3.45	2.11
		Std	1.23	20.25	3.24	23.03	6.29	2.98
	Altitude cost	Mean	13.97	59.84	14.81	16.73	72.31	14.71
		Std	9.45	25.02	8.55	8.49	27.82	6.73
Scenario 2	Total number		5914	932	3276	2737	836	4547
	Path length	Mean	994.83	1038.24	1005.14	1021.91	1103.56	1099.71
		Std	1.80	12.19	39.82	67.57	88.03	49.77
	Threat cost	Mean	3.18	5.39	35.21	7.24	13.95	14.98
		Std	4.72	7.12	9.70	6.55	9.77	45.44
	Smooth cost	Mean	2.43	23.37	6.61	12.66	49.41	13.51
		Std	1.33	15.73	9.12	23.84	41.08	26.23
	Altitude cost	Mean	13.46	104.17	18.67	18.61	150.63	31.39
		Std	14.63	35.21	19.52	19.90	64.15	28.59
Scenario 3	Total number		5872	370	1753	1286	175	889
	Path length	Mean	1001.38	1099.99	1018.22	1098.08	1075.35	1093.76
		Std	7.19	48.84	45.70	24.30	32.55	67.93
	Threat cost	Mean	4.30	7.20	6.01	12.92	39.56	27.51
		Std	3.61	9.91	5.31	6.47	17.73	11.34
	Smooth cost	Mean	5.65	60.69	12.08	39.99	169.31	31.59
		Std	17.32	52.21	11.38	20.38	53.62	32.95
	Altitude cost	Mean	22.20	229.83	31.66	63.20	208.63	69.35
		Std	18.35	44.62	19.67	28.50	65.69	41.25

**Table 12:** Wilcoxon rank-sum test results for task-level metrics and IGD under the three scenarios.

Scenario		Algorithm				
		MOCOA	NSGA-II	MOWOA	MOPSO	MOOOA
Scenario 1	path length	3.173 × 10^−11^ +	8.418 × 10^−2^ =	1.37 × 10^−3^ +	6.696 × 10^−11^ +	3.988 × 10^−4^ +
	threat cost	4.029 × 10^−1^ =	8.51 × 10^−1^ =	5.956 × 10^−1^ =	3.858 × 10^−1^ =	9.168 × 10^−1^ =
	smoothness cost	2.934 × 10^−5^ +	4.152 × 10^−2^ +	1.370 × 10^−5^ +	4.788 × 10^−8^ +	3.337 × 10^−2^ +
	altitude cost	1.411 × 10^−9^ +	9.47 × 10^−1^ =	1.171 × 10^−2^ +	1.613 × 10^−10^ +	2.062 × 10^−1^ =
	IGD	2.716 × 10^−11^ +	6.046 × 10^−7^ +	1.596 × 10^−7^ +	1.154 × 10^−10^ +	3.183 × 10^−6^ +
Scenario 2	path length	3.338 × 10^−11^ +	9.941 × 10^−3^ +	1.087 × 10^−10^ +	8.993 × 10^−11^ +	9.792 × 10^−10^ +
	threat cost	6.911 × 10^−3^ +	4.537 × 10^−7^ +	5.365 × 10^−3^ +	1.509 × 10^−2^ +	3.05 × 10^−2^ +
	smoothness cost	4.151 × 10^−4^ +	5.972 × 10^−1^ =	7.198 × 10^−3^ +	1.989 × 10^−9^ +	3.05 × 10^−2^ +
	altitude cost	8.485 × 10^−9^ +	6.283 × 10^−6^ +	3.034 × 10^−2^ +	3.825 × 10^−9^ +	1.437 × 10^−5^ +
	IGD	8.303 × 10^−11^ +	1.777 × 10^−10^ +	2.251 × 10^−2^ +	5.322 × 10^−11^ +	4.515 × 10^−8^ +
Scenario 3	path length	3.020 × 10^−11^ +	4.714 × 10^−5^ +	3.52 × 10^−7^ +	6.696 × 10^−11^ +	2.998 × 10^−9^ +
	threat cost	4.509 × 10^−5^ +	1.905 × 10^−4^ +	8.302 × 10^−6^ +	6.092 × 10^−11^ +	1.373 × 10^−10^ +
	smoothness cost	2.734 × 10^−10^ +	1.291 × 10^−5^ +	4.18 × 10^−7^ +	5.133 × 10^−12^ +	1.852 × 10^−4^ +
	altitude cost	3.338 × 10^−11^ +	8.883 × 10^−6^ +	7.506 × 10^−8^ +	3.020 × 10^−11^ +	1.031 × 10^−3^ +
	IGD	1.411 × 10^−11^ +	7.380 × 10^−10^ +	1.254 × 10^−7^ +	1.010 × 10^−8^ +	7.062 × 10^−5^ +
+/−/=		14/0/1	11/0/4	14/0/1	14/0/1	13/0/2

## Data Availability

All data in this paper are included in the manuscript. The MATLAB source code and experimental configurations supporting the results of this study are available at: https://github.com/bbdbqq/IML-MOWOA-UAV-Path-Planning (accessed on 19 June 2026).
